# A multistep (semi)-continuous biocatalytic setup for the production of polycaprolactone†

**DOI:** 10.1039/d3re00536d

**Published:** 2023-12-19

**Authors:** Alessia Valotta, Daniela Stelzer, Tamara Reiter, Wolfgang Kroutil, Heidrun Gruber-Woelfler

**Affiliations:** a Institute of Process and Particle Engineering, Graz University of Technology Inffeldgasse 13 8010 Graz Austria valotta@tugraz.at woelfler@tugraz.at; b Department of Chemistry, NAWI Graz, BioTechMed Graz, Field of Excellence BioHealth, University of Graz Heinrichstrasse 28 8010 Graz Austria

## Abstract

Biocatalysis has gained increasing importance as an eco-friendly alternative for the production of bulk and fine chemicals. Within this paradigm, Baeyer Villiger monoxygenases (BVMOs) serve as enzymatic catalysts that provide a safe and sustainable route to the conventional synthesis of lactones, such as caprolactone, which is employed for the production of polycaprolactone (PCL), a biocompatible polymer for medicinal applications. In this work, we present a three-step, semi-continuous production of PCL using an entirely biocatalytic process, highlighting the merits of continuous manufacturing for enhancing biocatalysis. First, caprolactone is produced in batch from cyclohexanol using a coenzymatic cascade involving an alcohol dehydrogenase (ADH) and BVMO. Different process parameters and aeration modes were explored to optimize the cascade's productivity. Secondly, the continuous extraction of caprolactone into an organic solvent, needed for the polymerization step, was optimized. 3D-printed mixers were applied to enhance the mass transfer between the organic and the aqueous phases. Lastly, we investigated the ring-opening polymerization of caprolactone to PCL catalyzed by *Candida antarctica* lipase B (CAL-B), with a focus on eco-friendly solvents like cyclopentyl-methyl-ether (CPME). Space–time-yields up to 58.5 g L^−1^ h^−1^ were achieved with our overall setup. By optimizing the individual process steps, we present an efficient and sustainable pathway for PCL production.

## Abstract 2

This work presents polycaprolactone (PCL) synthesis *via* BVMO-driven caprolactone production, followed by continuous monomer extraction and polymerization. Achieving high space–time yield, our approach offers a sustainable pathway for PCL production.

## Graphical abstract

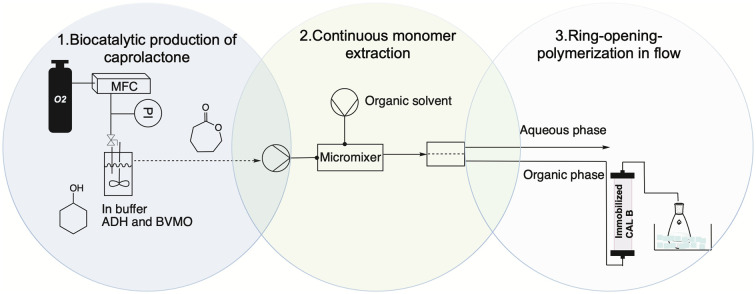

## Introduction

**Scheme 1:**
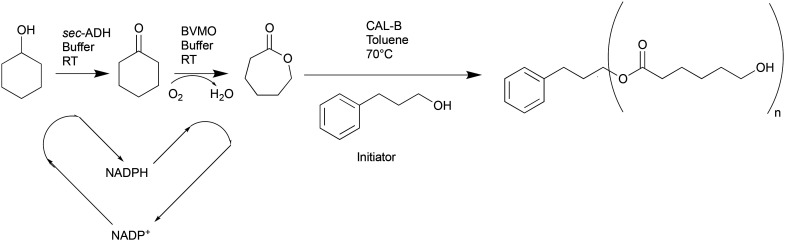
Scheme of the reaction cascade for the production of PCL presented in this work. First, cyclohexanol is converted to cyclohexanone by a *sec*-ADH with the aid of NADP^+^ as a cofactor, which is converted to NADPH. This fuels the second reaction in the cascade, which consists of the oxidation of cyclohexanone to caprolactone in the presence of molecular oxygen and catalysed by the BVMO enzyme. Caprolactone is further extracted and used as a feed for its ROP to PCL in the presence of 3-phenyl-propanol as an initiator.

Over the last few decades, biocatalysis has emerged as a promising and environmentally friendly technique for producing bulk and fine chemicals on both small and large scales.^1,2^ Biocatalysts have numerous advantages, such as high activity and specificity in mild conditions, which make them highly efficient and sustainable catalysts.^1–4^ Moreover, numerous enzymes could potentially substitute aggressive and dangerous chemicals in the route to produce valuable compounds.^5^ A group of enzymes that has attracted a lot of interest in this regard is Baeyer Villiger monoxygenases (BVMO).^6^ These enzymes can catalyse the oxidation of (cyclic) ketones in the presence of molecular oxygen by inserting one atom of oxygen in a C–C bond, generating water as a by-product.^6^ An example is the production of caprolactone, which is used as a monomer for the production of polycaprolactone (PCL). This biodegradable and biocompatible polymer has been successfully used in biomedical applications for sutures, wound dressing, and tissue engineering.^7^ Caprolactone is traditionally produced from cyclohexanone using peracetic acid in stoichiometric amounts, which renders the process potentially hazardous and environmentally harmful.^8,9^ Therefore, the biocatalytic production of caprolactone by BVMO has been suggested in literature as a greener alternative to this process.^10–13^ Since BVMOs are NADPH-dependent enzymes, a coenzymatic reaction cascade for the production of lactones involving an alcohol dehydrogenase (ADH) and a BVMO was first proposed in 1991 to efficiently solve the issue of cofactor recycling for this system.^14^ Since then, numerous works have reported improved versions of this cascade, starting from the readily available bulk chemical cyclohexanol, and the activity of the two enzymes has been thoroughly studied to understand and overcome the intrinsic product and substrate inhibition that limits the BVMO activity.^9,11,15^ Reactor engineering has also been implemented recently to further increase the cascade's productivity. The reaction could be scaled up in fed-batch mode, and different aeration modes have been tested (*e.g.*, membrane aeration or direct sparging).^16,17^ Motivated by the high efficiency of this cascade, researchers have thought to combine this step with the production of PCL. This polymer can be generally produced by chemical or biocatalytic routes, using in this case the lipase B from *Candida antarctica* (CAL-B).^18^ CAL-B has been widely explored as a ring-opening polymerization (ROP) catalyst since it is highly active in organic solvents, such as toluene, which is the solvent of choice for the ROP of caprolactone. Moreover, using CAL-B allows to obtain polymers with good yield and mechanical properties and without residual amounts of metal-based catalysts, and it furthermore requires lower reaction temperatures than other catalysts.^16,19^ Lastly, CAL-B is commercially available in immobilized form at various suppliers and shows high operational stability.^20^ Therefore, the combination of the biocatalytic production of caprolactone with its subsequent polymerization has been presented in literature, either as a one-pot process in water or as a sequence of a fed-batch process for monomer production followed by separate batch steps to isolate the monomer and initiate its polymerization.^10,16^ Despite the promising applications, performing most of the steps to the final product in batch slows down the production of the final product and causes losses along the production line. Moreover, the connection of the monomer production to the ROP step is limited by the incompatibility between the BVMO and the organic solvent needed to achieve an appreciable conversion of caprolactone. Spatial compartmentalization could be applied to increase compatibility between the two steps and speed up the process.^21,22^ An approach in this direction has been introduced in batch by Gröger *et al.*,^23^ who have developed a PDMS membrane system that is permeable to caprolactone and allows its transfer from an aqueous phase, where the biocatalytic oxidation takes place to the organic phase, where immobilized CAL-B was used to initiate the polymerization. This system offered good conversions and achieved appreciable amounts of PCL oligomers; however, it still had some limitations in the low starting concentration of caprolactone (100 mM). Another possibility to increase the compatibility between different steps in a biocatalytic process, which has not been applied to this system yet, is to switch to continuous flow processes.^22^ In fact, by confining multiple steps into separate but interconnected continuous unit operations, it is possible to overcome the incompatibility of biocatalytic reactions to organic solvents when performing the process in one pot. Moreover, it is also possible to achieve and uphold the optimal process conditions for each step in the system and to avoid the need for isolation of intermediates, thereby reducing errors and losses along the production line.^22,24–27^ Utilizing reactors with inner dimensions in the mm range for these applications also allows for improved heat and mass transfer and better control of the different unit operations, resulting in more reliable processes.^28^ In this work, we aim to present a novel approach for a three-step, semi-continuous production of PCL to show the advantages that continuous manufacturing offers to increase the speed and productivity of biocatalytic processes. Therefore, the multistep reaction cascade shown in Scheme 1 has been developed to produce PCL. It consists of a first module, which has already been presented in literature,^15^ where first cyclohexanol is oxidized to cyclohexanone by a secondary alcohol dehydrogenase (*sec*-ADH) from *Lactobacillus brevis*, which then undergoes oxidation to caprolactone catalysed by a BVMO from *Acinetobacter calcoaceticus*. The first reaction is fuelled by NADP^+^ as a cofactor, which is then converted to NADPH and made available for the O_2_/NADPH-dependent oxidation of cyclohexanone, thereby being efficiently recycled. For this first step, different reactor concepts have been tested to intensify the reaction by exploring different parameters and aeration systems, reporting here the first comparison in literature. However, due to the long reaction times needed to achieve an appreciable conversion and high concentrations of caprolactone, the reaction is difficult to transfer to flow since changing the temperature and pressure to intensify the reaction is limited by the possible deactivation of the enzyme. Therefore, in this work we have setup an efficient batch reaction that allowed for the complete conversion of up to 200 mM of cyclohexanol, with similar productivity to what was reported in literature.^15–17^ Then, the first step batch served as a feed for a continuous process, which included a second step to extract the monomer from the buffered aqueous solution into an organic solvent in flow. For this purpose, a Zaiput flow extractor SEP-10 was used, and different mixing systems, including a range of in-house designed 3D printed mixers, were investigated to identify the contacting system that allows for ideal mass transfer between the two phases. The extraction is followed by the subsequent ROP of caprolactone catalyzed by commercially available immobilized CAL-B in the presence of 3-phenyl-propanol as an initiator, as suggested in literature.^29^ The biocatalytic polymerization of PCL has already been tested in flow microreactors, starting from commercially available caprolactone, with good monomer conversion and polymerization degree.^29–32^ However, to our knowledge, this is the first time a semi-continuous process has been designed to produce PCL, starting from the *in situ* biocatalytic generation and continuous flow extraction of the monomer. The ROP of caprolactone has been further investigated and adapted to this application to employ the knowledge from the literature to the process at hand. Moreover, different organic solvents were considered to substitute toluene to make the polymerization a greener step. In this regard, cyclopentyl-methyl-ether (CPME) has been chosen as both reaction and extraction solvent due to its medium polarity, high boiling point, extremely low water miscibility and high stability compared to other green solvents such as 2-methyl-tetrahydrofuran.^33^ In this work, the optimization of all three steps will be discussed separately, showing all the parameters involved in the process design. Finally, the overall process will be presented, which has been built and tested under different conditions to improve the conversion of caprolactone and its polymerization to provide an efficient and sustainable approach to the production of PCL.

## Results and discussion

### Characterization of the biocatalytic production of caprolactone

The *sec*-ADH and BVMO enzymes used in this work have been presented in a precedent work.^15^ However, while in the original publication they were used in purified form, in this work both enzymes were utilized as lyophilized cell-free extract (CFE) to reduce the time and resources otherwise spend for enzyme purification. Therefore, preliminary experiments were conducted to determine the ratio of the two enzymes to achieve an optimal production of caprolactone, as shown in Table 1. For these tests, 200 mM of cyclohexanol and 2 mM of NADP^+^ dissolved in 2 mL of buffer (100 mM sodium phosphate buffer at pH 8) were charged in 8 mL vials equipped with a septum for enabling the oxygen flow *via* a needle. The oxygen was delivered at a flow rate of 5 mL min^−1^*via* a mass flow controller (MFC) connected to an oxygen bottle, and the reaction was carried out at room temperature and at a stirring speed of 350 rpm. Samples were taken every 0,1,2,4 and 24 hours and were measured by GC-FID to determine the cyclohexanol conversion and caprolactone yield. The ratios of enzymes tested and the relative results in terms of conversion, yield and specific activity are reported in Table 1. It was found that using 15 mg mL^−1^ of BVMO CFE and 2.5 mg mL^−1^ of ADH gave the highest yield of caprolactone. Therefore, the enzyme ratio was kept constant for following experiments. Moreover, it was noticed that the exact conversion and yield as for the purified enzymes were achieved for our case, indicating that the enzyme as CFE had comparable activity reported previously.^15^

**Table 1:** Set of investigated ratios of *sec*-ADH to BVMO enzymes with obtained values for the conversion of cyclohexanol, the yield of caprolactone, and the BVMO's specific activity. All runs were carried out at room temperature, with 5 mL min^−1^ of oxygen and a starting concentration of cyclohexanol of 200 mM and 2 mM of NADP^+^

Ratio ADH : BVMO [g L^−1^]	Conversion [%]	Yield [%]	Specific activity [U g_BVMO_^−1^]
30 : 30	100	62	5.54
5 : 30	100	88	8.06
2.5 : 15	100	98	12.34

**Fig. 1:**
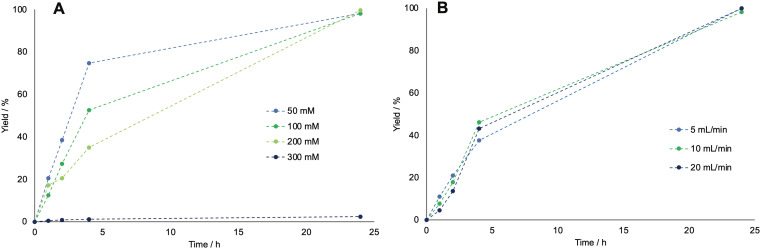
Time course of the amount of caprolactone for varied cyclohexanol concentration and varied oxygen flow rates, as determined by GC-FID. A: GC-yield of caprolactone for starting concentrations of cyclohexanol ranging from 50 to 300 mM, for runs using 5 mL min^−1^ for the oxygen flowrate. B: GC-yield of caprolactone for varied oxygen flow rates, for runs using 200 mM starting concentration of cyclohexanol. All runs were carried out at room temperature and with 2 mM of NADP^+^, 15 mg mL^−1^ of BVMO and 2.5 mg mL^−1^ ADH enzyme. The dotted lines have been drawn to guide the eye.

Other parameters were explored in batch to further characterize the system and intensify the reaction. First, different concentrations of cyclohexanol were tested, as shown in Fig. 1A ranging from 50 to 300 mM, to test the limits of the BVMO's activity.

The results indicated increased inhibition of the enzymes with increasing starting cyclohexanol concentrations, which could not be increased above 200 mM without high losses in enzymatic activity. Second, the effect of increasing the flow rate up to 20 mL min^−1^ was tested, though it did not drastically improve the reaction rate, therefore the flow rate was kept to 5 mL min^−1^ (as shown in Fig. 1B).

**Fig. 2:**
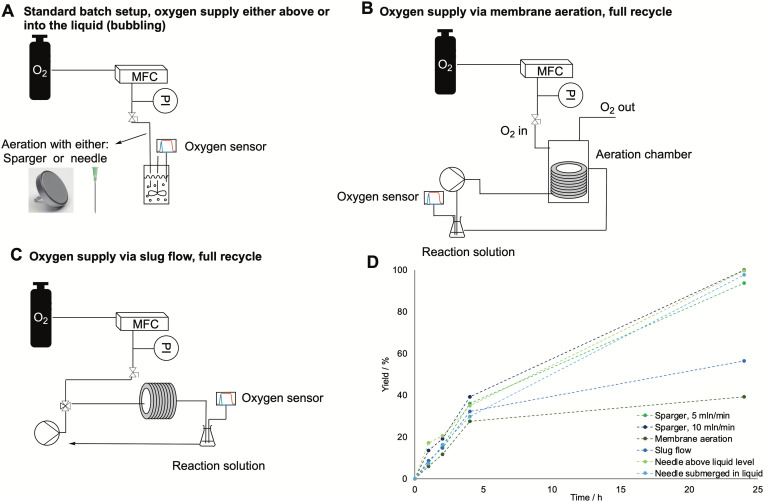
A: Batch setup for testing the needle and the sparger as oxygen flow systems. B: Membrane aeration system. C: Slug flow system. D: Course of the yield of caprolactone with time for the different oxygen supply systems, as determined by GC-FID. All runs were carried out at room temperature, with 5 mL min^−1^ of oxygen and a starting concentration of cyclohexanol of 200 mM, 2 mM of NADP^+^, 15 mg mL^−1^ of BVMO and 2.5 mg mL^−1^ ADH enzyme. The dotted lines have been drawn to guide the eye.

In biocatalytic oxidations catalyzed by BVMO the oxygen supply is pivotal to intensify the reaction, since molecular oxygen is one of the reactants. However, its solubility in buffered solutions is very low, and to avoid enzyme deactivation it is only possible to vary the temperature and the pressure within small ranges, *i.e.*, around ambient conditions, which do not positively affect the overall oxygen solubility.^34^ Therefore, we decided to test multiple oxygen supply systems to improve the mass transfer and intensify the reaction to scale it up for polymer production. The modes of oxygen supply tested in this work are shown in Fig. 2. First, a common lab needle with an internal diameter of 0.8 mm was used to supply oxygen either above or into the reaction solution. It was tested in comparison to a metal flat sparger (porosity 2–10 μm), also submerged in the liquid (see Fig. 2A). In the last two cases, antifoam SE15 (0.2 v%) was added to avoid foaming. Then, two different flow modes were tested: membrane aeration and slug flow (see Fig. 2B and C respectively). In the first case, the setup was built similarly to what was proposed in literature. The oxygen was supplied from a MFC connected to an oxygen bottle, which flowed into a glass aeration chamber where a silicon coil was inserted, through which the reaction solution was pumped in a recycling mode by a peristaltic pump. The oxygen flow rate was 5 mL min^−1^, while the pump flow rate was 2.5 mL min^−1^. For the slug flow, the oxygen and the reaction solution came in contact in a T-junction, after which a slug flow was induced and the resulting solution was pumped through a PVC tube in recycle. In this case, both the oxygen and the reaction solution flow rate were set to 2.5 mL min^−1^ to induce the formation of evenly distributed slugs.

As the results in Fig. 2D show, neither the sparger nor the flow setups drastically increased the reaction rate. To investigate this behavior further, the *k*_L_*a* values for each configuration were tested by inserting an oxygen sensor in the system and determining the mass transfer coefficient *via* the gassing out method (see experimental section for details on the *k*_L_*a* determination). The obtained *k*_L_*a* values are shown in Table 2, and for all aeration systems they were found to be in the expected range for lab-scale (below 100 mL) bioreactors.^35,36^ The results also show that the mass transfer rates are in line with the obtained reaction outcomes, with the best aeration mode being the needle either above or inside the liquid. However, differences in the *k*_L_*a* values are limited in a small range and the same amount of dissolved oxygen is reached at equilibrium (for the oxygen curves, see the ESI†). Therefore, neither the efficiency of oxygen feeding to the system nor the size of the gas bubbles affect the amount of dissolved oxygen, therefore, the reaction remains kinetically rather than mass-transfer limited.

**Table 2:** *k*_L_ *a* values obtained for different aeration systems

Oxygen supply system	*k*_L_ *a* [h^−1^]
Needle above liquid	8.06
Needle in liquid	10.6
Sparger 5 mL min^−1^	5.82
Membrane aeration	4.65
Slug flow	5.12

**Fig. 3:**
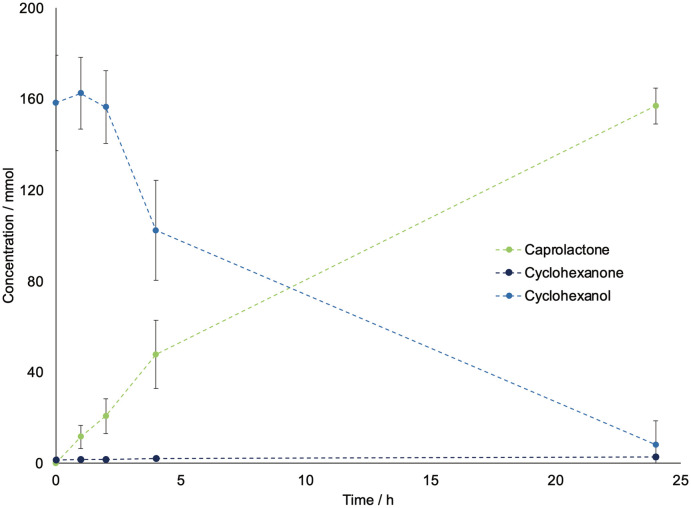
Progress curve for cyclohexanol, cyclohexanone, and caprolactone concentrations in a 20 mL batch, as determined by GC-FID. Error bars correspond to standard deviations from independent triplicates. The reactions were carried out at room temperature, with 5 mL min^−1^ of oxygen and a starting concentration of cyclohexanol of 200 mM, 2 mM of NADP^+^, 15 mg mL^−1^ of BVMO and 2.5 mg mL^−1^ ADH enzyme. Since the standard deviation for the concentration of cyclohexanone was always below 1%, this is not shown in the graph.

Due to the long time needed to achieve an appreciable conversion and the limited intensification possibilities, this step was carried out in batch to produce the feed for the subsequent polymerization. Therefore, we have proceeded to further scale-up the batch from 2 mL to 20 mL, with the setup shown in Fig. 2A using the needle as oxygen supply system. Fig. 3 shows the progress curve for the 20 mL experiments, which achieved a concentration of caprolactone up to 170 mM and comparable conversion and yield to that obtained for the 2 mL reaction, resulting in a linear scale-up without productivity losses and good reproducibility (based on independent triplicates).

Also, the productivity showed similar trends to other works reported in literature.^16,17^ Due to these encouraging results, we did not investigate the utilization of a fed batch reactor, as it was shown in literature to not influence significantly the overall reaction time.^16,17^

### Investigation of the polymerization step

**Fig. 4:**
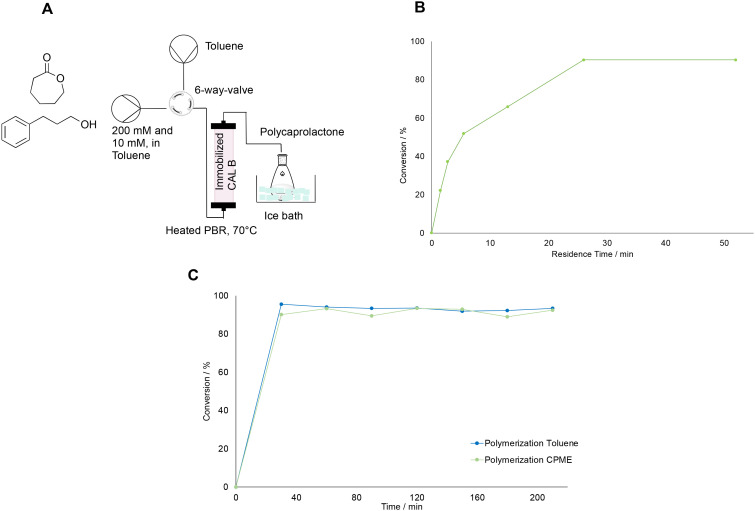
A: Setup used for investigating the ring opening polymerization of caprolactone catalysed by immobilized CAL-B. B: Change in the conversion of caprolactone with increasing residence time, as measured with GC-FID. C: Results of the polymerization experiments with either toluene or CPME as solvents, carried at 0.1 mL min^−1^ over the course of 2 hours. All runs were carried out at 70 °C and with a starting concentration of caprolactone of 200 mM and 10 mM of the initiator 3-phenyl propanol. The lines in the graphs are for visual guidance only, not a model fit.

The polymerization was carried out in a similar reactor to that proposed in literature.^29^ The setup had to be adapted to the limited initial concentration of around 200 mM determined by the biocatalytic step. Therefore, the choice of flow rate was first carried out by determining the conversion of caprolactone for different residence times. For this purpose, the setup as shown in Fig. 4A was built. It consisted of two syringe pumps, one filled with the monomer and the initiator, and one filled with toluene for flushing the reactor in between experiments. Caprolactone and 3-phenyl propanol were used in a molar ratio of 20 : 1, therefore the concentration was 200 and 10 mM, respectively, following the ratio suggested in literature.^29^ The pumps were connected to the system *via* a 6-way valve, which allowed to switch to the washing step after an experimental run rapidly. The valve was then attached to the tubular reactor, which consisted of a PTFE tube with a 1.58 mm internal diameter filled with 150 mg of immobilized CAL-B (on Immobead 150, 1800 U g^−1^ from Sigma-Aldrich) and kept at 70°. This temperature had been chosen, since extensive kinetic studies performed in literature in similar fixed bed reactor conditions suggest that above 70 °C the reaction rate of caprolactone polymerization does not increase with increasing temperatures.^30^ Moreover, this temperature was also reported by other groups using fixed bed reaction processes, and served as guidelines for this work.^29,32^ The reactor outlet was then connected to a flask filled with cold methanol in an ice bath to induce the precipitation of the polymer. The polymer was fully dissolved in the solvent before precipitation in methanol, and the reactor exhibited no signs of clogging throughout the process. Consequently, pressure control was unnecessary, as the pressure drop appeared constant during the reaction, aligning with observations from other fixed-bed enzymatic polymerization reactors documented in the literature.^29,30,32^ With this setup, flow rates between 0.05 to 2 mL min^−1^ (corresponding to residence times between 52 to 1.5 min) were tested to identify the residence time for which the highest conversion of caprolactone (as determined by GC-FID) could be achieved. Therefore, for each experiment, the reactor was first flushed with toluene until it was filled, then the valve was switched and the monomer solution with the initiator was pumped into the system at the desired flow rate. Samples were collected for GC-FID at the reactor's outlet until steady state was achieved, typically after three times the residence time for each point. Then, the reactor was flushed again with solvent with at least five reactor volumes and then a new flowrate was set and a new run was carried out as before. Experiments were carried out starting from the highest to the lowest flow rate, and the results [Fig. 4B] showed, that the conversion increased rapidly in the range between 1.5 and 15 minutes of residence time. Then, the conversion reached an optimum for flow rates between 0.2 to 0.05 mL min^−1^. Therefore, the flow rate for the further reaction experiments was set to 0.1 mL min^−1^. At this optimal flow rate, a long polymerization run was further carried out with both toluene and a green solvent, CPME. This solvent was chosen since it proved to extract caprolactone from water and is a greener alternative to toluene, which has been the most used solvent for the polymerization of caprolactone. An exception are solvent-free polymerizations, which would not be a viable solution for our biocatalytic setup. The polymerization in the longer runs was carried out for 2 hours, and samples were taken every 30 minutes and measured by GC-FID to determine the conversion of caprolactone. Fig. 4C shows that the conversion achieved was around 93% for both solvents. Consequently, they were further tested in the whole setup as viable solvent alternatives. Other green solvents, such as ethyl acetate and 2-methyl-tetrahydrofuran, were not considered since they showed to inhibit the polymerization in some preliminary tests (data not shown).

### Optimization of the extraction module

**Fig. 5:**
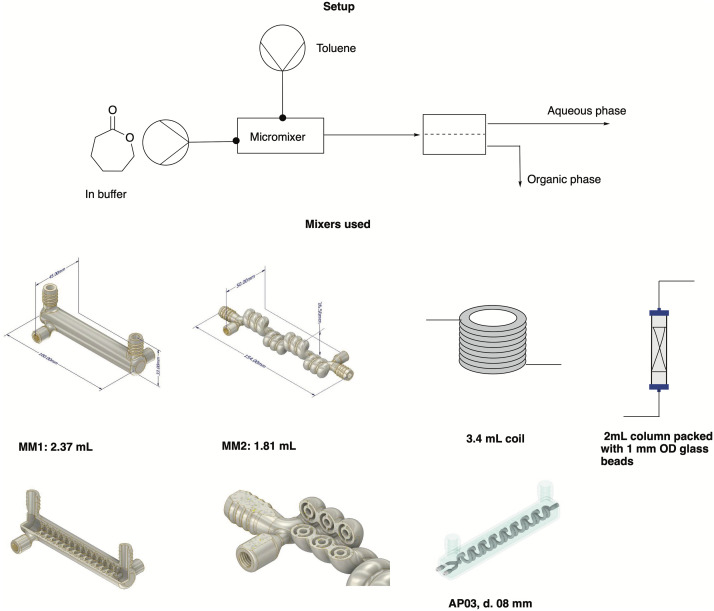
Graphical scheme of the setup used for the extraction experiments, with a closer look at the mixers tested in this work. For a close-up of MM1 and MM2, see the ESI.†

A continuous extraction system was built and tested to extract the monomer for the polymerization in flow, with a Zaiput SEP-10 extractor equipped with a hydrophobic membrane (OB-900) chosen according to the manufacturer's recommendation. Different mixing systems were included to identify the optimal contacting time and mixing behavior between the two phases to achieve a high mass transfer from the aqueous to the organic phase. As a parameter to assess the extraction efficiency, the *K*-value was used, defined as (1):1
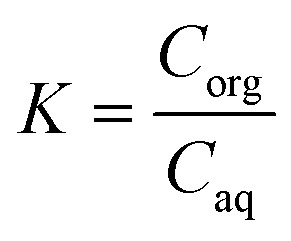
where *C*_org_ is the concentration of the analyte in the organic phase and *C*_aq_ in the aqueous phase. The setup and the different mixers used are shown in Fig. 5, and the results are summarized in Table 3. All experiments were carried out using 1 : 1 ratios of both phases (toluene and buffer with 200 mM caprolactone) and a flow rate of 0.1 mL min^−1^, which was selected to ensure enough residence time in the polymerization reactor and to ensure good phase contacting in the mixer. The used mixers were chosen to compare different internal volumes and mixing efficiencies and their effect on the extraction.

**Table 3:** Summary of the extraction experiments' results for different systems in both batch and flow modes. All experiments were carried out using 1 : 1 ratios of both phases (toluene or CPME and buffer with 200 mM caprolactone) and for the flow runs, a flow rate of 0.1 mL min^−1^ was used for each pump

Solvent	Mixer	Concentration organic phase [mM]	Concentration aqueous phase [mM]	*K*-Value
Toluene	Metal mixer 1	146.8	48.4	3.03
	Metal mixer 2	89.6	97.9	0.91
	Packed column	93.6	118.2	0.79
	Metal mixer AP03	94.7	97.1	0.98
	Coil	149.9	62.03	2.42
CPME	Metal mixer 1	90.6	88.07	1.03
CPME	Batch	82.2	56.2	1.46
Toluene	Batch	106.2	35.3	3

The metal mixers used in this work were designed in-house within our group, and 3D printed at Anton Paar GmbH by selective laser melting. The goal was to design mixers that allowed for good phase contacting by introducing bends and hinders in the flow, enabling the formation of secondary flows while keeping the internal volume low and the mixer compact. The AP03 reactor was already presented in a previous publication,^37^ while the new metal mixer 1 (MM1) and metal mixer 2 (MM2) are introduced for the first time in this work. MM1 consists of a cylinder where the reaction channel runs in a spiral at the inner surface. MM2 is instead characterized by a helicoidal channel whose direction is changed every three turns to improve mixing. Both mixers have an internal diameter of 1 mm, a length of 100 mm for MM1 and 154 mm for MM2 and a volume of 2.37 and 1.81 mL respectively. Both have been characterized by residence time distribution experiments (see the ESI† for details on the RTD). According to the RTD results, the MM1 mixer showed on average lower backmixing than the MM2, due to the flow being more chaotic in the MM1 mixer, causing increased mixing by chaotic advection of the incoming streams. The two further mixers tested were a simple PTFE coil, 226 cm long and with an internal diameter of 0.8 mm, and a 2 mL stainless steel HPLC column filled with 2 g of 1 mm glass beads.

With the mixers at hand, volumes from 0.3 to 3.4 mL were tested, which resulted in residence times ranging from 1.5 to 17 minutes. The performed experiments showed that the lower the backmixing, the better the separation. Therefore, the coil reactor and the MM1 were the best-performing mixers in the list. Moreover, a volume between 2.37–3.4 mL (or a residence time between 12–17 minutes) seemed to provide enough contacting time to achieve an extraction efficiency comparable to a reference system in batch (see Table 3). Therefore, the system was built with MM1 to keep the residence time low while obtaining an appreciable mass transfer efficiency. To apply the same approach to CPME, the extraction efficiency of this solvent was tested in batch and in flow to determine the *K*-value. While this was not as high as for the extraction in toluene, the solvent was still considered a viable possibility for the whole flow setup.

### Combination of all steps in one flow setup

**Fig. 6:**
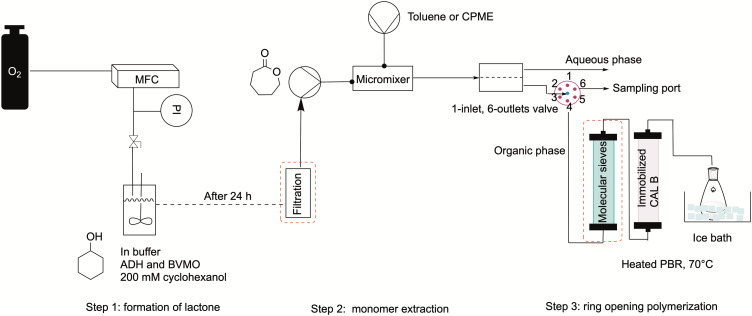
Schematic view of the overall setup used to produce PCL starting from the biocatalytic production of the monomer caprolactone. Marked in red dashed boxes are the filtration and solvent drying units, which were inserted in the process in some experiments and tested to assess their effect on the polymerization. The final process did not include these two units.

The whole setup was combined as shown in Fig. 6. First, the monomer was produced overnight in a 20 mL stirred tank with the configuration shown in subsection 2.1. Furthermore, the need for a filtration system before the extraction step was tested, and different filters were compared to a non-filtered feed. The feed solution containing the monomer was then pumped into the MM1, where it came in contact with the organic solvent, either toluene or CPME. Then, the two phases were separated in the SEP-10, after which a 6-way valve was mounted to allow a switch between one port for sample taking for determining the concentration in the organic phase, and one for feeding the solution to the polymerization reactor. This was kept at 70 °C and was filled with 150 mg immobilized CAL-B. In some tests, the organic phase was dried in a column filled with molecular sieve before entering the reactor. Finally, the reactor's outlet stream was dropped into a flask filled with 50 mL of ice-cold methanol to initiate the polymer precipitation. Samples were taken at the six-way valve after the extractor and at the reactor's outlet for GC-FID to determine the conversion of caprolactone. The outlet solution collected in the flask was treated *in vacuo* to evaporate the solvent and isolate the polymeric product for testing with ^1^HNMR or GPC measurements, to determine the number average and weight average molecular weight respectively (*M*_n_ and *M*_w_).

**Fig. 7:**
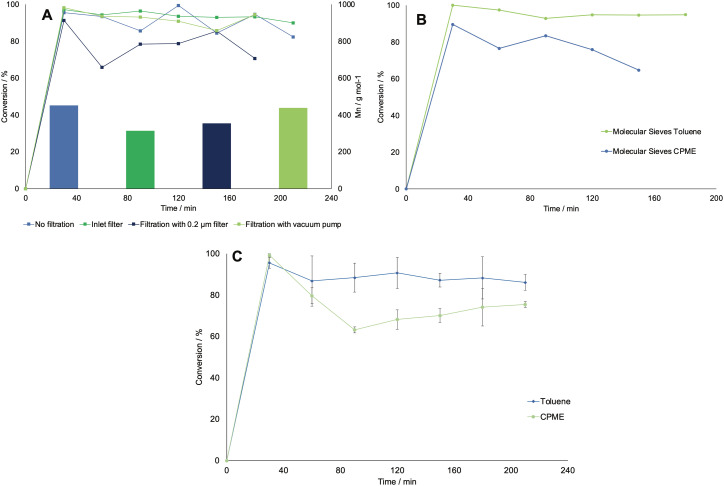
A: Progress curves for the conversion of caprolactone in different systems where the feed solution was filtered with different methods and extracted in toluene. The bars in the graphs also show the number average molecular weight *M*_n_ obtained *via*^1^H NMR for each experiment. B: Progress curves for the conversion of caprolactone in a flow system which included the presence of a column filled with molecular sieves. Both toluene and CPME were tested as solvents in this setup. C: Progress curves for the conversion of caprolactone in the reproducibility tests for the final setup, tested both in toluene and CPME, which did not include the filtration nor the molecular sieves units (error bars correspond to standard deviations from three triplicates). All runs were carried out at 70 °C and with 170 mM of starting concentration of caprolactone and 10 mM of 3-phenyl propanol as initiator, and a flow rate of 0.1 mL min^−1^ was set for each pump. The lines are for visual guidance only, not a model fit.

First, the effect of using different filters was investigated. This was deemed necessary, as the feed solution was dense due to the presence of debris from the CFEs, which could block the small inner channels of the flow equipment. The occurrence of a viscous solution was also deemed responsible of decreasing the lifetime of the SEP-10 membrane, which had to be changed every 3rd experiment. First, the solution was filtrated *in vacuo* and in batch mode over a filter with a porosity of 10–16 μm. Then, in another run, the solution was pumped in flow through an HPLC pre-column filter, which had a porosity of 2 μm. In the next run, the solution was again filtered in batch with a 0.2 μm PTFE membrane filter. As a comparison, a run without filtration was carried out. All experiments were carried only out using toluene as organic phase, as the choice of solvent was not deemed to influence the filtration in this stage. Fig. 7A shows the results in terms of conversion (determined by GC-FID) and *M*_n_ (determined by ^1^H NMR) for all the tested systems. The conversion of caprolactone was maintained at a similar average value as for the not-filtered solution, except for the inline filter run, where the conversion is slightly lower. However, the results indicate lower *M*_n_ values for the filtered systems, which were generally below 400 g mol^−1^, compared to the non-filtered system, with which a value of 454 g mol^−1^ was achieved. A possible explanation was found when looking at the ^1^H NMR analysis of the isolated caprolactone from the first step (see Fig. S5†). The spectrum showed the presence of oligomers after the biocatalytic cascade, therefore the loss of these oligomers during the filtrations step could have caused a lower efficiency of the ROP.

In an effort to improve the low *M*_n_ obtained, the utilization of molecular sieves in flow was tested to determine the effect of solvent dryness on the polymerization, as it was reported in literature to influence the activity of CAL-B.^30,32^ Therefore, an HPLC column filled with 2 g of molecular sieves (10 w/v% of the overall processed volume) was included in the setup to dry the organic stream coming from the SEP-10 into the ROP reactor. The setup was then tested, with the feed from the biocatalytic step without prior filtration, for both toluene and CPME. Karl-Fischer titration measurements were carried out by taking 1 mL samples of solvent before and after the molecular sieves column and measured them right away to avoid errors. The results indicated that it was possible to decrease the water content in toluene from 0.039 w% to 0.008 w%, while for CPME the water content could only decrease from 0.638 w% to 0.116 w%, due to the partial solubility of water in this solvent. As shown in Fig. 7B, in terms of conversion, little to no change was found in the setup with molecular sieves for both solvents, indicating that the kinetics of CAL-B was not largely influenced by the solvent dryness in this range. Once again, to compare the setup with and without molecular sieves, the *M*_n_ was determined by ^1^H NMR. Also GPC measurements were carried out to further validate the results and determine the *M*_w_ and the polydispersity index *Đ*, given as:2
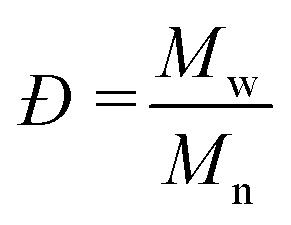
where *M*_w_ and *M*_n_ are the weight- and number average molecular weights as determined by GPC. The *Đ* was calculated to assess the goodness of the polymer distribution, as values closer to one gives a narrow molecular weight dispersity. The overview of the obtained results for both setups is shown in Table 4. Results showed that for both solvents the *M*_n_ was not positively influenced by the presence of molecular sieves. However, when it came to the *M*_w_, a higher amount of longer chained polymers was detected in the case of the polymerization in CPME, indicated by an increase from 415 to 454 g mol^−1^, mirrored by an increase in *Đ* from 1.15 to 1.3. The polymerization in toluene was instead not affected by the addition of molecular sieves, and the value for *M*_w_ was 532 g mol^−1^ for the basic setup and 527 g mol^−1^ for the setup with molecular sieves. The reason is most probably the difference in water content before and after the molecular sieve column, which was significantly lower for toluene compared to CPME. However, to ensure reproducibility and better compare the efficiency of the two solvents, the setup was further replicated without a filter nor molecular sieves for both toluene and CPME. The results showed good conversion and low standard deviation (see Fig. 7C), however, the values for the *M*_w_ were still limited due the low starting concentration of caprolactone.

**Table 4:** Summary of the results for the polymerization experiments performed with the whole setup. All runs were carried out at 70 °C and with the presence of 3-phenyl propanol as initiator in a 20 : 1 molar ratio (referred to caprolactone), and a flow rate of 0.1 mL min^−1^ was set for each pump

Solvent	Starting concentration [M]	Conversion^b^ [%]	*M*_n_ [g mol^−1^]		*M*_w_ c [g mol^−1^]	*Đ* [—]	Isolated polymer [g]	STY^d^ [g L^−1^ h^−1^]
			^1^H NMR	GPC				
Toluene	2	60	7269	7345	14 874	2.02	2.1	911.4
	0.17	90	452	407	532	1.3	0.2	58.5
	0.17^a^	95	418	380	527	1.39	0.19	55.1
CPME	2	38	3864	3918	7294	1.86	1.55	673.9
	0.17	77	389	356	415	1.15	0.1	40.3
	0.17^a^	78	393	348	454	1.3	0.14	28.9

a These experiments were carried out in a setup with the addition of a column filled with molecular sieves.

b Referred to caprolactone, obtained by GC-FID.

c Determined by GPC.

d Calculated considering the internal volume of the polymerization reactor.

As a proof of concept to assess the applicability of our setup to higher initial concentrations of caprolactone, the polymerization step was tested with a starting concentration of 2 M of commercially available caprolactone in both toluene and CPME. In this case, the *M*_w_ was 14 874 g mol^−1^ for toluene, 28 times higher than what achieved at 170 mM of caprolactone. For CPME, the *M*_w_ was 7294 g mol^−1^, which was 17 times higher than the value achieved at 170 mM of caprolactone. These results indicate that the degree of polymerization is non linearly dependent on the starting concentration of caprolactone, as already suggested in literature.^18^ With these results, the designed flow process proved to be applied successfully to higher starting concentrations of caprolactone, therefore the limiting step remains the productivity of the biocatalytic Baeyer–Villiger oxidation step.

Finally, to assess the productivity of the different setups, the space–time-yield (STY) was determined:3
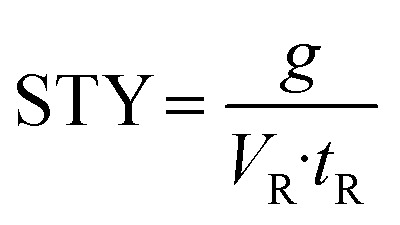
where *g* is the amount of polymer isolated at the end of the experiment, *V*_R_ is the internal volume of the polymerization reactor (in L) and *t*_R_ is the operating time (in h). As the values in Table 4 show, the STY was higher for the setup with toluene compared to that with CPME, with a value of 58.5 g L^−1^ h^−1^*versus* 40.3 g L^−1^ h^−1^. The setup with molecular sieves gave a similar value for the STY in both cases. With the scaled-up polymerization, a productivity of 911.4 g L^−1^ h^−1^ was determined for toluene and 673.9 g L^−1^ h^−1^ for CPME. These results indicate that the process, both in small and large concentration scales, could produce appreciable amounts of PCL, which make it promising for future applications.

## Conclusion

In this work, we have successfully designed and tested the first semi-continuous entire biocatalytic process for the production of PCL. We have started with the optimization of the first module, namely the production of caprolactone in a biocatalytic cascade. In this regard, we have chosen the *sec*-ADH and BVMO cascade proposed in literature,^15^ starting from cyclohexanol and with an in-built NADPH recycle. To improve the system, we have tested both the effect of the substrate inhibition and the flow rate of the oxygen and saw that we could only convert up to 200 mM of cyclohexanol, and changing the oxygen flow rate did not positively affect the reaction rate. Therefore, we went on to test different aeration modes, which also did not improve the reaction rate, as the reaction is kinetically limited. These results aligned to the slow reaction rate of BVMO reported in literature.^10,15,17^ Moreover, so far no application showed the possibility to increase the biocatalytic conversion of cyclohexanol to higher final concentrations of caprolactone, which hinders the application of this biocatalytic cascade in industry. A possible future solution would be to continue with the research in protein engineering to design more stable and active BVMOs, which would be less product-inhibited. Such achievement would also enable the switch from batch to flow, thereby allowing to design a fully continuous process which could potentially have a higher STY and achieve more appreciable amounts of PCL in a shorter time.

The application of a 3D printed mixer combined with an extraction module in flow proved to be a successful approach to efficiently isolate the monomer prior to its polymerization. We have no doubt that in the future the SEP-10 could also be successfully applied to higher concentration of caprolactone, as a higher concentration difference between the organic and the aqueous phase would result in a higher gradient which would ensure efficient mass transfer between the two phases. An issue that still needs to be addressed though is the lifetime of the membrane, which is low in the presence of dense solutions. Immobilizing the ADH and BVMO enzymes would make the filtration of the feed not necessary as the solution would be free of debris. Few studies have reported successful BVMO immobilization methods,^6,38,39^ and so far the increased mass transfer limitations have hindered the productivity of these systems. As a result, the reaction times to achieve a comparable conversion to the free enzyme system are too long for it to be a viable alternative, at least until an improved immobilization system is developed. Another improvement that could be implemented to increase the economic viability of the extraction process would be the recovery of the monomer from the aqueous phase after extraction, to possibly re-use the monomer for polymerization. On industrial scale, continuous solvent removal is a well-established unit operation (*e.g.* by using falling film evaporators). On lab scale, this could be done by removing the water in batch, but to improve the productivity of the process, an flow evaporation unit could be tested. An interesting prototype for a flow evaporator has been developed in the research group of Steven Ley, which has been successfully applied to a multitude of solvents, including water.^40^

Regarding the polymerization, with our approach high values of STY were achieved for both toluene and CPME, with values up to 58.5 and 40.3 g L^−1^ h^−1^ respectively. However, the polymer length achieved with the whole setup was limited to around 500 g mol^−1^, but still higher to what achieved in literature in one pot,^10^ demonstrating the advantage of using a flow system. The main problem though is that the system is very diluted since the starting concentration is limited by the first step. For systems with low initial polymer concentrations, the polymerization degree is of course lower.^18,41^ Therefore the only way to improve the setup would be to produce more caprolactone in the first step, as the production of PCL by ring-opening polymerization with an increased monomer concentration proved to be successful and gave polymers with a molecular weight up to 14 874 g mol^−1^ and a STY up to 911.4 g L^−1^ h^−1^. A solution in this regard would be to partially or totally remove the solvent by evaporation, *e.g.* by using a flow evaporator as mentioned before, to have the polymerization in an almost solvent-free environment.^40^ Such approach could improve the resulting *M*_w_ and it has already been applied in batch for the enzymatic ROP catalysed by CAL-B,^16^ however, the implementation of an evaporation unit will need to be carefully assessed as it increases the energy demand of the process. Nevertheless, we have also shown how implementing a greener solvent such as CPME could provide a valid alternative to toluene as an ROP solvent. At low starting concentrations of caprolactone, only limited difference in the value for the *M*_w_ compared to toluene was recorded (415 compared to 532 g mol^−1^), and comparable STY was achieved (58.5 compared to 40.3 g L^−1^ h^−1^). However, for the experiment at 2 M of caprolactone, only half of the *M*_w_ achieved with toluene was obtained with CPME. We believe that by performing a more specific optimization of the reaction conditions for CPME (flowrate, catalyst amount, effect of the solvent dryness) it would be possible to improve these results in the future.

Finally, it would be of great interest to apply the shown setup to other substrates, starting from naturally derived compounds such as terpene and terpenoids. One terpenoid of particular interest is carvone, which can be hydrogenated to carvomenthone and dihydrocarvone which are suitable substrates for BVMOs and further ring-opening polymerization. Few studies exist in literature,^42–44^ however, we believe that combining the process presented in this work with these natural feedstocks would be of great interest to produce biocompatible polymers in a greener way.

In conclusion, we believe that with this work we have shown that by applying systematic optimization and using engineering tools, such as continuous flow, it is possible to intensify biocatalysis and open the way to more productive and sustainable processes.

## Experimental

### General

All chemicals and solvents were purchased from Sigma Aldrich and TCI Chemicals unless stated otherwise and used as received.

### Enzyme expression and lyophilization

Enzymes were produced according to the following procedures.^15^ For expression of the *sec*-ADH, *E. coli* C43(DE3) cells transformed with the pEG180 plasmid were grown at 30 °C and 120 rpm in a 50 mL Sarstedt tube containing 10 mL LB/Amp (final concentration 100 mg mL^−1^ of ampicillin) medium overnight. LB/Amp medium (700 mL; 100 mg mL^−1^ ampicillin) in 2 L baffled shaking flasks was inoculated with 5 mL of the overnight culture (ONC). These cultures were shaken at 37 °C and 120 rpm until OD600 reached 0.8. Anhydrotetracycline (70 μL of a 2 mg mL^−1^ stock solution; final conc. 0.2 μg mL^−1^) was added. The culture was shaken overnight at 20 °C and 120 rpm. Cells were harvested by centrifugation (8000 rpm, 11 500 g, 20 min, 4 °C) and washed with potassium phosphate buffer (50 mM, pH 7.5). For the BVMO, the enzyme was overexpressed in *E. coli* NEB 10-beta by using terrific broth (TB) medium containing ampicillin (100 mg mL^−1^) in baffled flasks. TB/Amp was inoculated using 1% ONC and incubated at 37 °C /120 rpm until OD_600_ was 0.6–1 (*ca.* 3 h), followed by induction using l-arabinose (0.02% w/v, 100 μl from 0.5 g mL^−1^ stock) and further expressed at 24 °C during 36 h. Cells were harvested by centrifugation (8000 rpm, 20 min, 4 °C) and washed with Tris-HCl (50 mM, pH 7.5). For both enzymes, after harvesting the cells were resuspended in washing buffer and sonicated with a Branson Sonifier 250 with horn 102C and micro tip (pulse mode, 1 s on, 4 s off, 5 min pulse time, 30% amplitude). Cell suspension was then centrifuged at 15 000 rpm, 4 °C for 20 min and the supernatant (CFE) was lyophilized overnight using Christ Alpha 1–4 LSC basic at −56 °C and 0.08 mbar.

### Procedure for the tests for the biocatalytic production of caprolactone

The first preliminary tests for determining the effect of enzyme ratio, substrate concentration, and oxygen flow rate on the biocatalytic production of caprolactone were conducted as follows. The necessary amounts of both enzymes (2.5 mg mL^−1^ of *sec*-ADH and 15 mg mL^−1^ of BVMO) were weighted in an 8 mL glass vial equipped with a septum to add the oxygen through a needle. Then, 2 mM of NADP^+^ (3 mg for the 2 mL scale) was added with 100 mM sodium phosphate buffer at pH 8 (1.958 μL in a typical experiment) and vortexed vigorously to dissolve the enzyme. Then, cyclohexanol was added (*e.g.*, 42 μL for a 200 mM experiment), and the total reaction volume was 2 mL. The oxygen was supplied *via* a mass flow controller connected to a pressurized gas bottle, then added through a needle (i.d. 0.8 mm) into the system. The flowrate was set to 5 mL min^−1^ for most experiments, the stirring rate was kept at 350 rpm, and the reactions were carried out at room temperature. Samples of 200 μL were taken after 0, 1, 2, 4 and 24 hours, each time extracted with 600 μL of ethyl acetate, dried over magnesium sulfate, and then centrifuged before measurement. A similar procedure was applied to the reaction scale-up, where a 50 mL flask equipped with a multiport screw cap was used to be filled with 20 mL of reaction solution (prepared analogously to what was described before, *i.e.*, 420 μL of cyclohexanol and 19.58 mL of buffer).

For the tests with the different oxygen supplies, the used procedures were as follows. For the sparger experiments, tests were carried out in the same setup as for the 20 mL batch, with the difference of the sparger instead of the needle as oxygen supply and with the addition of 4 μL (0.2 v%) of antifoam SE15. For the tests in the membrane aeration setup, 20 mL of the reaction solution were prepared as described above, then were kept on a stirring plate and pumped in recycle at 2.5 mL min^−1^ through a 2 m coil made of a thin silicon tube (0.5 mm thickness) and inserted in a glass chamber were oxygen was flowed through at a rate of 5 mL min^−1^. Samples were taken from the outlets of the silicon tube at the same time intervals of the batches, and measured as described above. For the slug flow experiments, the 20 mL reaction solution was stirred and pumped at a flowrate of 2.5 mL min^−1^ in recycle through the system. The solution came in contact with oxygen (flowing at 2.5 mL min^−1^) at a T-junction, were slug flow was induced and allowed to pump through a 2 m PVC coil. Samples were taken as for the membrane aeration setup.

### Procedure for the *k*_L_*a* measurements

*k*_L_ *a* measurements for the different oxygen supply systems were carried out using the dynamic gassing out method.^45^ Briefly, the reaction vessel was charged with 20 mL of reference medium (100 mM sodium phosphate buffer pH 8). Then the vessel was tightly closed with a screw cap, which presented fittings for a robust oxygen probe from Pyroscience GmbH, a PT100 temperature sensor and a needle to flush through with either argon or oxygen. The oxygen sensor was connected to a phase fluorimeter FireSting (also from Pyroscience GmbH) to collect the sensor data, which were displayed in the python-based Pyroscience Workbench software. The vessel (and the tubings for the slug flow and membrane aeration setups) was first purged with argon to remove all traces of oxygen inside the liquid and the system. Once the oxygen sensor, which was submerged in the liquid, gave a value close to 0, the gas supply was switched to oxygen and the experiment was performed as during the reaction experiments. The gradual increase of oxygen concentration in the liquid was recorded in the Pyroscience Workbench software until a steady value was reached. The *k*_L_*a* was determined by direct measurement of the rate of increase in dissolved oxygen concentration, after it was lowered by passing dry argon gas (oxygen free), through the system. The dynamic response of the process has been described by the following equation:4
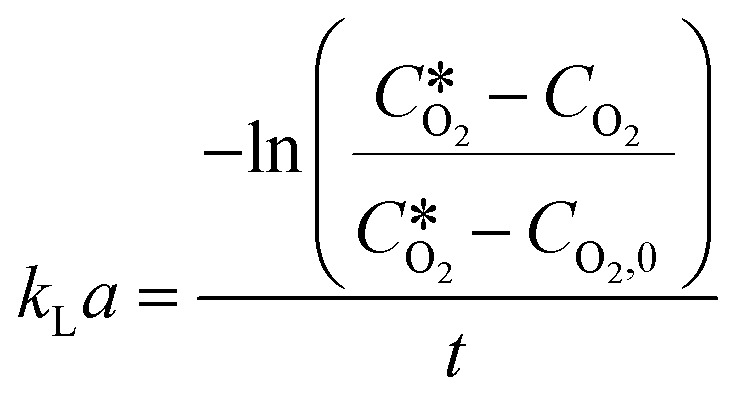
where *C*_O_2_,0_ is the oxygen concentration in the liquid phase initially (time (*t*) = 0) and 
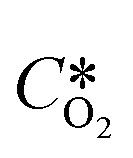
 is the oxygen concentration at equilibrium and *C*_O_2__ is the dissolved oxygen concentration at time *t*. By plotting the first term of the fraction over time, it is possible to determine the *k*_L_*a* value as the slope of the obtained curve by linear regression.

### Extraction tests in continuous flow

To investigate the continuous extraction of caprolactone from the water phase, the following procedure was used to test different mixers. Two syringe pumps, one filled with toluene and one with the caprolactone solution in buffer were connected *via* standard 1/32′′ i.d. PTFE capillaries to a T-junction, which was connected to the mixer's inlet. Then, the solution entered the Zaiput SEP-10 separator, after which the aqueous and organic phase were collected on the two opposite sides of the membrane. For this system, an OB-900 hydrophobic membrane was used as supplied by Zaiput. For each tested mixer, the *K*-value was used as an indicator to determine the efficiency of the extraction and it was calculated as the ratio between the concentration in the aqueous phase over the concentration in the organic phase, as determined by GC-FID. 300 μL of the organic phase were diluted with 300 μL of the organic solvent of choice and vortexed prior to measurement. The aqueous phase (200 μL of sample) was extracted with ethyl acetate (600 μL), dried over magnesium sulphate and then centrifuged prior to measurement.

### Polymerization tests in continuous flow

The investigation of the polymerization process in flow was adapted from the procedure of Adhami *et al.*^29^ A syringe pump was filled with 20 mL of solvent, while the other contained a solution containing caprolactone (200 mM or 2 M) dissolved in either toluene or CPME. Then, the initiator 3-phenylpropanol was added (either 10 or 100 mM depending on the starting concentration). A 0 sample of 300 μL was taken from the solution, diluted with 300 μL of the same solvent in a GC vial. The pumps were then connected to a six-way-valve, which was connected to the reactor *via* standard fittings. The reactor was previously filled with 150 mg of immobilized CAL-B (on Immobead 150, 1800 U g^−1^ from Sigma-Aldrich) and plugged with cotton at both ends to prevent the particle bed from moving. The reactor was further submerged in a water bath set to 70 °C. The reactor was first washed with only solvent, then, the six-way valve was switched and the monomer solution was pumped through the system. Samples were taken every 30 minutes at the reactor's outlet and prepared as done for the 0 sample. The samples were taken right at the reactor's outlet and measured with GC-FID. The output of the reactor was collected in a 100 mL round bottom flask filled with 50 mL of methanol and placed in an ice bath to promote the precipitation of the polymer. At the end of the experiment, the solvent in the flask was removed using vacuum filtration or evaporation under vacuum in case of lower monomer concentrations, to be further measure by ^1^H NMR or GPC.

### Procedure for the overall process

Firstly, a stirred tank was charged with 300 mg of BVMO and 50 mg of ADH enzymes. Then, buffer (19.58 mL) was added, and the solution was shaken well to allow enzyme dissolution. Afterwards, cyclohexanol was added (200 mM, 420 μL) under strong magnetic stirring, a 0 sample was taken, then the oxygen line was connected to the flask, the flow rate was set to 5 mL min^−1^ and the stirring to 350 rpm. The oxygen was supplied *via* a needle right above the liquid, as shown previously. 200 μL samples were taken after 1, 2, 4 and 24 hours and extracted with 600 μL EtOAc for GC detection. After 24 h, the solution was pumped *via* a syringe pump either through a filter or directly into a metal mixer, where it came in contact with the extraction solvent (either CPME or toluene), which contained the desired amount of initiator (10 mM of 3-phenylpropanol). The phases were then separated in a Zaiput SEP-10 extractor equipped with a hydrophobic membrane (OB-900). Samples of the aqueous phase were taken right at the aqueous phase port of the extractor and treated as done for the 1st batch step (200 μL of sample extracted with 600 μL EtOAc). Samples of the organic phase were taken every hour at a 6-way-valve placed after the extractor, which allowed to switch between the reactor and the sampling port when necessary. The organic phase was then pumped into the polymerization reactor, filled with 150 mg of catalyst beads. The reactor outlet was dropped into a 50 mL ice-cold methanol and isolated following the procedure from the polymerization experiments. Samples for GC-FID were taken also at the outlet of the reactor every 30 minutes. As done for the polymerization tests, the samples of the organic phase (300 μL) were diluted with the same amount of solvent (300 μL) prior to measurement.

### GC analysis

The samples (200 μL) from the first biocatalytic step and the aqueous phase after the extractor was extracted with ethyl acetate (600 μL). The organic phase was dried over anhydrous MgSO_4_ and centrifuged before measurement. The polymerization samples (300 μL) were diluted with the same solvent of the reaction (300 μL). GC analysis was performed with a Perkin Elmer Clarus 500 equipped with an Optima-5 MS capillary column (Machery-Nagel, 30.0 m × 320 μm ID, 0.25 μm) with a flame ionization detector (FID). For details on the GC methods, see the ESI.†

### Karl Fischer titration measurements

To determine the water content of the solvents, samples were taken in the system at different conditions. 1 mL samples of the solvent after the extractor and after the molecular sieves were taken to determine the amount of water in the samples *via* Karl-Fischer titration.

The liquid sample was transferred *via* syringe from the HPLC-vial on the scale for differential weighing and then directly injected into the measuring cell of the coulometric Karl Fischer titrator. The titrator model used is an SI Analytics TitroLine® 7500 KF trace without diaphragm, the reagent used was “Merck CombiCoulomat fritless”.

### ^1^H NMR analysis

Proton (^1^H) NMR measurements were carried out for both the isolated caprolactone from the first step and for determining the number average molecular weight *M*_n_ of polycaprolactone at different process conditions following the procedure from literature.^29,32^ To isolate caprolactone, the reaction solution of a 20 mL batch was extracted 3 times with 20 mL ethyl acetate and concentrated *in vacuo* to remove the solvent and isolate caprolactone. The samples from the flow experiments were also concentrated *in vacuo* to remove the reaction and precipitation solvents (toluene or CPME and methanol respectively), the obtained product being a cotton-like fibrous material. At least 20 mg of sample were then diluted in 700 μL of deuterated chloroform and then analysed with a using a Bruker Avance III 300 MHz spectrometer. The obtained .fid data was evaluated using the MestreNova software. For details on the assigned NMR shifts and further calculations, see the ESI.†

### GPC analysis

In order to obtain the values for *M*_n_ and *M*_w_ more accurately, gel permeation chromatography (GPC) measurements were carried out for the final flow experiments, to cross-check with the NMR data. First, a sample of the polymer was dissolved in THF containing BHT as internal standard, to achieve a final concentration of 5 mg mL^−1^. The sample was left to sit for one day, then it was filtrated with a 0.45 μm PTFE membrane filter prior to injection in the system. The instrument used was a GPC from Shimadzu, equipped with a LC-20 AD pump, a SIL-20 AC HT autosampler and both a RID 20A refractive index detector and a SPD-40 UV-vis detector. The system was equipped with a pre-column MZ-Gel SDplus 50 × 8 mm, then two columns were installed, both MZ-Gel SDplus Linear 5 μm, 300 × 8 mm. For each sample, 100 μL were injected in the system and analysed at a flowrate of 1 mL min^−1^ over the course of 35 minutes. The molecular weight distributions obtained for each measured sample are reported in the ESI.†

## Author contributions

Conceptualization, A. V., W. K. and H. G. W.; methodology, A. V. and D. S.; investigation, D. S. and A. V.; formal analysis, D. S. and A. V.; resources, H. G. W., T. R. and W. K.; data curation, D. S. and A. V.; writing—original draft preparation, A. V.; writing—review and editing, all authors; visualization, D. S. and A. V.; supervision, H. G. W. and W. K.; project administration, A. V.; funding acquisition, H. G. W. and W. K. All authors have read and agreed to the published version of the manuscript.

## Conflicts of interest

There are no conflicts to declare.

## Supplementary Material

RE-009-D3RE00536D-s001

## References

[cit1] Wu S., Snajdrova R., Moore J. C., Baldenius K., Bornscheuer U. T. (2021). Angew. Chem., Int. Ed..

[cit2] Woodley J. M. (2019). Appl. Microbiol. Biotechnol..

[cit3] Faber K. , Biotransformations in Organic Chemistry, Springer International Publishing, 2018

[cit4] Buchholz K. , Kasche V. and Bornscheuer U. T., Biocatalysts and Enzyme Technology, Wiley-VCH Verlag & Co. KGaA, 2012

[cit5] Benítez-Mateos A. I., Roura Padrosa D., Paradisi F. (2022). Nat. Chem..

[cit6] Delgove M. A. F., Valencia D., Solé J., Bernaerts K. V., De Wildeman S. M. A., Guillén M., Álvaro G. (2019). Appl. Catal., A.

[cit7] Mondal D., Griffith M., Venkatraman S. S. (2016). Int. J. Polym. Mater. Polym. Biomater..

[cit8] Kayser M. M. (2009). Tetrahedron.

[cit9] Staudt S., Bornscheuer U. T., Menyes U., Hummel W., Gröger H. (2013). Enzyme Microb. Technol..

[cit10] Schmidt S., Scherkus C., Muschiol J., Menyes U., Winkler T., Hummel W., Gröger H., Liese A., Herz H.-G., Bornscheuer U. T. (2015). Angew. Chem., Int. Ed..

[cit11] Mallin H., Wulf H., Bornscheuer U. T. (2013). Enzyme Microb. Technol..

[cit12] Bučko M., Gemeiner P., Schenkmayerová A., Krajčovič T., Rudroff F., Mihovilovič M. D. (2016). Appl. Microbiol. Biotechnol..

[cit13] Leisch H., Morley K., Lau P. C. K. (2011). Chem. Rev..

[cit14] Willetts A. J., Sandeya H., Shipston N. F. (1991). J. Chem. Soc..

[cit15] Sattler J. H., Fuchs M., Mutti F. G., Grischek B., Engel P., Pfeffer J., Woodley J. M., Kroutil W. (2014). Angew. Chem., Int. Ed..

[cit16] Scherkus C., Schmidt S., Bornscheuer U. T., Gröger H., Kara S., Liese A. (2016). ChemCatChem.

[cit17] Srinivasamurthy V. S. T., Böttcher D., Engel J., Kara S., Bornscheuer U. T. (2020). Process Biochem..

[cit18] Labet M., Thielemans W. (2009). Chem. Soc. Rev..

[cit19] Engel J., Cordellier A., Huang L., Kara S. (2019). ChemCatChem.

[cit20] Remonatto D., Miotti Jr. R. H., Monti R., Bassan J. C., de Paula A. V. (2022). Process Biochem..

[cit21] Stepankova V., Bidmanova S., Koudelakova T., Prokop Z., Chaloupkova R., Damborsky J. (2013). ACS Catal..

[cit22] Liu Y., Liu P., Gao S., Wang Z., Luan P., González-Sabín J., Jiang Y. (2021). Chem. Eng. J..

[cit23] Wedde S., Rommelmann P., Scherkus C., Schmidt S., Bornscheuer U. T., Liese A., Gröger H. (2017). Green Chem..

[cit24] Žnidaršič-Plazl P. (2021). Curr. Opin. Green Sustainable Chem..

[cit25] Benítez-Mateos A. I., Contente M. L., Roura Padrosa D., Paradisi F. (2021). React. Chem. Eng..

[cit26] De Santis P., Meyer L.-E., Kara S. (2020). React. Chem. Eng..

[cit27] Chaichol P., Weeranoppanant N. (2023). React. Chem. Eng..

[cit28] Plutschack M. B., Gilmore K., Seeberger P. H. (2017). Chem. Rev..

[cit29] Adhami W., Bakkour Y., Rolando C. (2021). Polymer.

[cit30] Kundu S., Bhangale A. S., Wallace W. E., Flynn K. M., Guttman C. M., Gross R. A., Beers K. L. (2011). J. Am. Chem. Soc..

[cit31] Huang W., Zhu N., Liu Y., Wang J., Zhong J., Sun Q., Sun T., Hu X., Fang Z., Guo K. (2019). Chem. Eng. J..

[cit32] Bhangale A. S., Beers K. L., Gross R. A. (2012). Macromolecules.

[cit33] de Gonzalo G., Alcántara A. R., Domínguez de María P. (2019). ChemSusChem.

[cit34] Delgove M. A. F., Elford M. T., Bernaerts K. V., Wildeman S. M. A. D. (2018). Org. Process Res. Dev..

[cit35] Kostov Y., Harms P., Randers-Eichhorn L., Rao G. (2001). Biotechnol. Bioeng..

[cit36] Kirk T. V., Szita N. (2013). Biotechnol. Bioeng..

[cit37] Maier M. C., Valotta A., Hiebler K., Soritz S., Gavric K., Grabner B., Gruber-Woelfler H. (2020). Org. Process Res. Dev..

[cit38] Solé J., Brummund J., Caminal G., Schürman M., Álvaro G., Guillén M. (2019). Appl. Catal., A.

[cit39] Bučko M., Schenkmayerová A., Gemeiner P., Vikartovská A., Mihovilovič M. D., Lacík I. (2011). Enzyme Microb. Technol..

[cit40] Deadman B. J., Battilocchio C., Sliwinski E., Ley S. V. (2013). Green Chem..

[cit41] Kobayashi S. (2009). Macromol. Rapid Commun..

[cit42] Lowe J. R., Martello M. T., Tolman W. B., Hillmyer M. A. (2011). Polym. Chem..

[cit43] Wilbon P. A., Chu F., Tang C. (2013). Macromol. Rapid Commun..

[cit44] Messiha H. L., Ahmed S. T., Karuppiah V., Suardíaz R., Ascue Avalos G. A., Fey N., Yeates S., Toogood H. S., Mulholland A. J., Scrutton N. S. (2018). Biochemistry.

[cit45] Suresh S., Srivastava V., Mishra I. (2009). J. Chem. Technol. Biotechnol..

