# Supplementary material for: A multistep (semi)-continuous biocatalytic setup for the production of polycaprolactone
Source: React Chem Eng. 2023 Dec 19;9(3):713–27. doi: 10.1039/d3re00536d (PMC10903532; doi:10.1039/d3re00536d)
Supplement: RE-009-D3RE00536D-s001 [file RE-009-D3RE00536D-s001.pdf]

Electronic Supplementary Material (ESI) for Reaction Chemistry & Engineering.
This journal is © The Royal Society of Chemistry 2023

A multistep (semi)-continuous biocatalytic setup for the production of polycaprolactone
Supporting information

Alessia Valotta,[a,*] Daniela Stelzer,[a] Tamara Reiter,[b] Wolfgang Kroutil,[b] Heidrun Gruber-Woelfer,[a,*].

[a]*Institute of Process and Particle Engineering, Graz University of Technology, Inffeldgasse 13, 8010 Graz, Austria.*

[b]*Department of Chemistry, University of Graz, NAWI Graz, BioTechMed Graz, Field of Excellence BioHealth, Heinrichstrasse 28, 8010 Graz, Austria.*

[*] *Corresponding authors: valotta@tugraz.at (A.V.); woelfler@tugraz.at (H.G.W.).*

Contents

Contents 1
1. Pictures of the setups used in this work 2
2. Design and manufacturing 3D printed metal mixers 3
3. Sensor readout of the $k_L a$ determination experiments 5
4. Characterization of the residence time distribution in the 3D printed metal mixers 5
4.1 Testing of possible hydrolysis of caprolactone in MM1 7
5. Characterization of the residence time distribution in the packed tubular reactor 8
6. GC method and calibration curves 9
7. ^{1}H-NMR spectra 11
7.1 Calculation of the number-average molecular weight (M_n) 13
8. Polymer characterization via GPC 13
9. References 16

1. Pictures of the setups used in this work

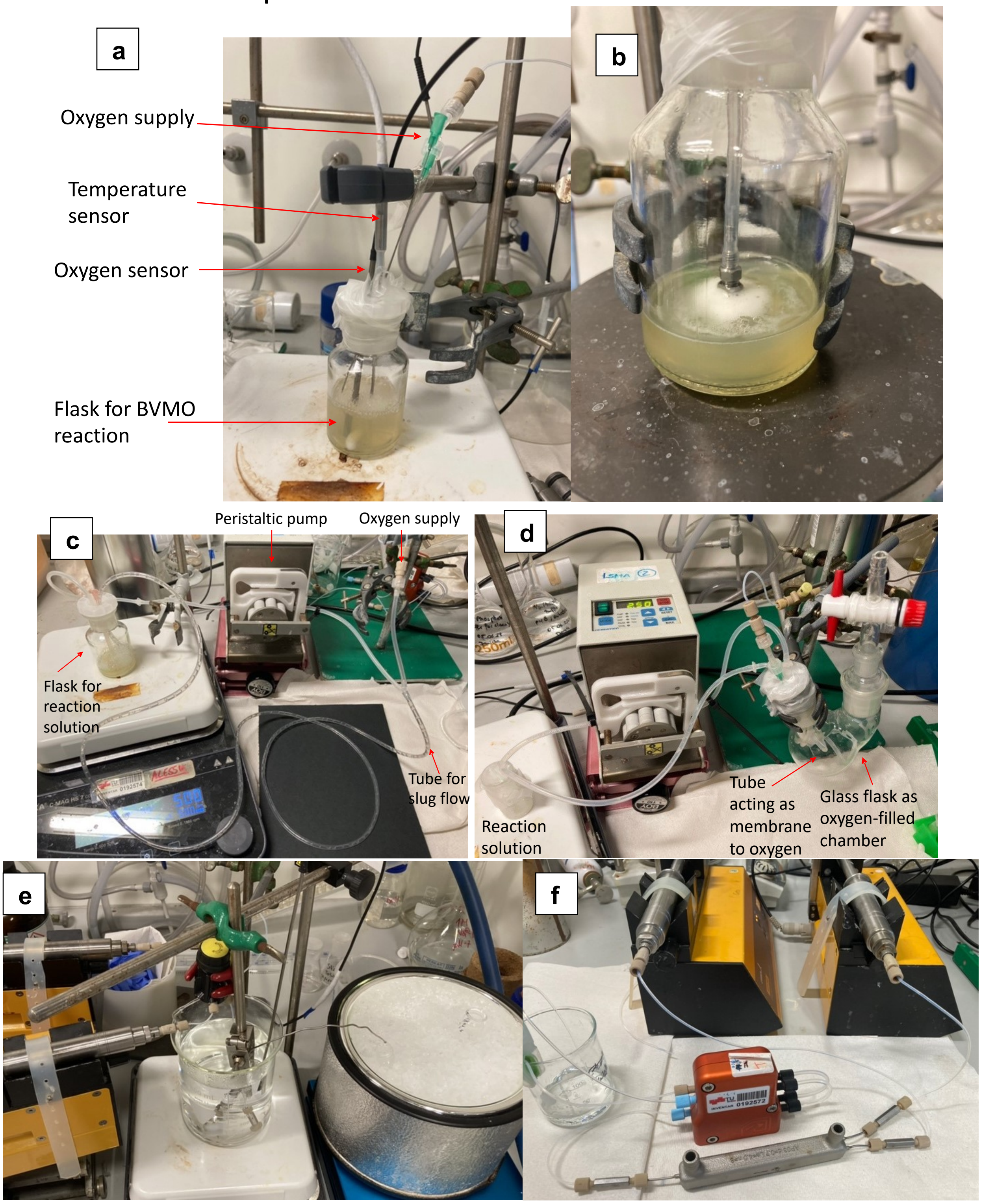

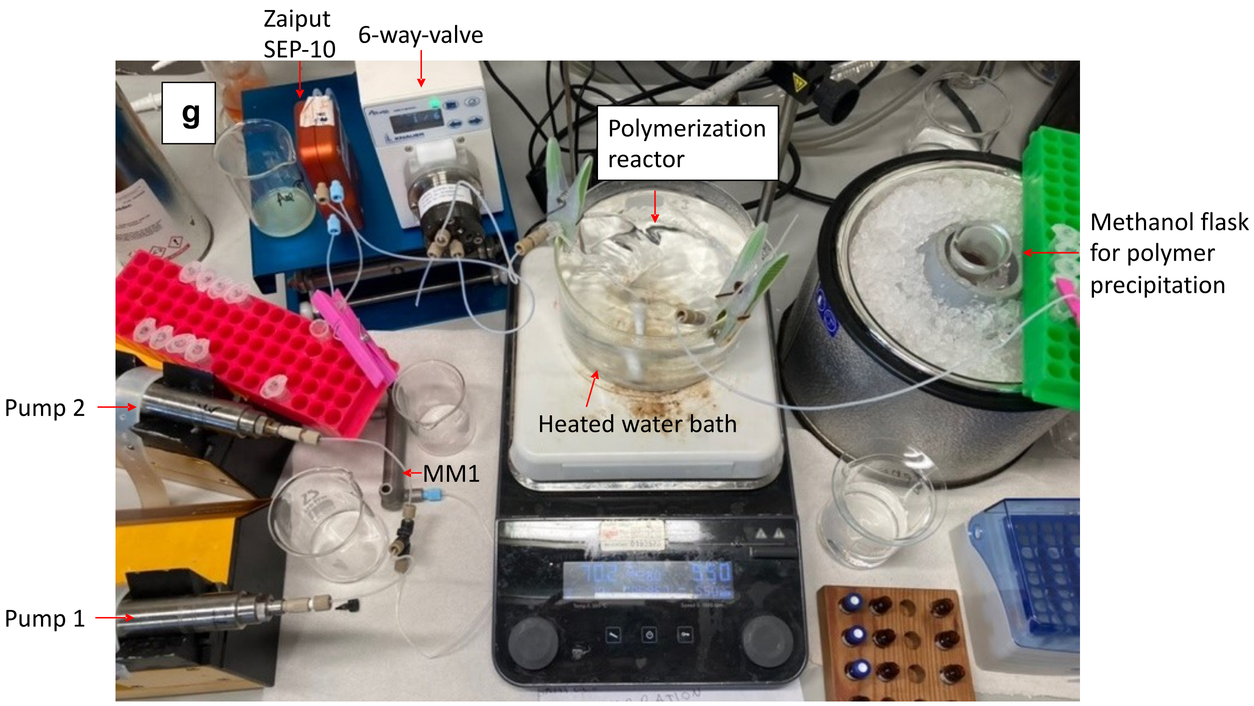

Figure S1 – (a) Figure of the 20 mL stirred flask used for the biocatalytic cascade production of caprolactone. The oxygen was supplied by a needle above the liquid level. (b) Same as for a, but in this case the oxygen was supplied via a flat sparger. (c) Slug flow setup. (d) Membrane aeration setup. (e) Setup for only the polymerization step. (f) Setup for testing the extraction efficiency. (g) Final overall setup for the multistep production of caprolactone.

2. Design and manufacturing 3D printed metal mixers

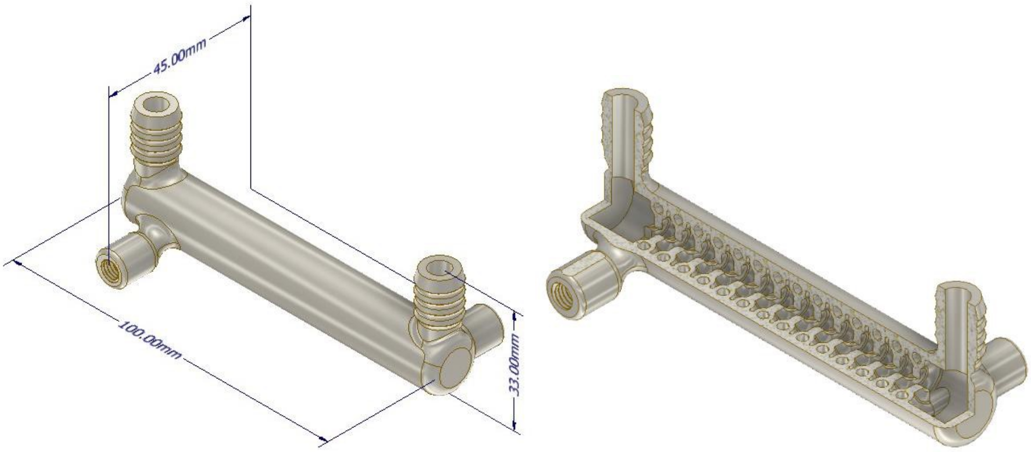

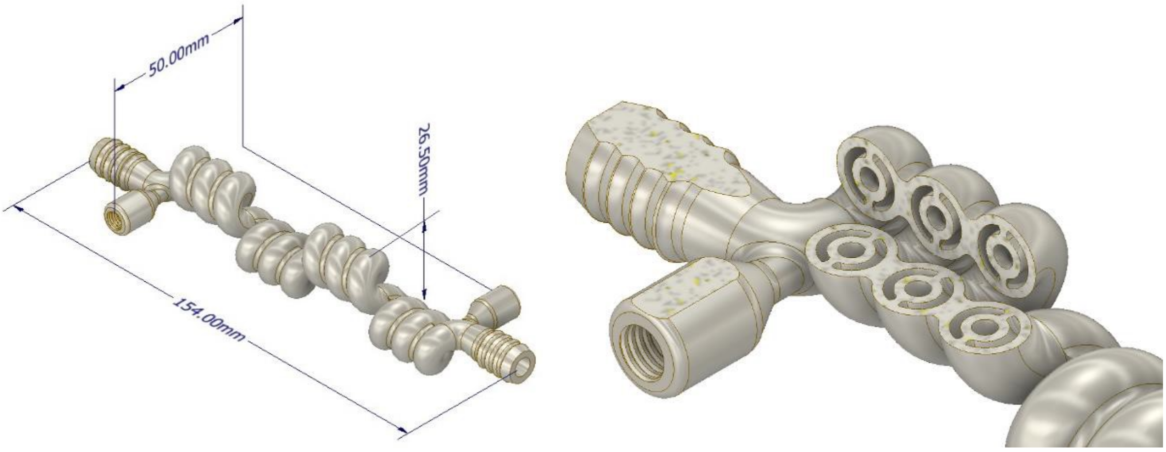

Figure S2 – Close-up at the structures of MM1 and MM2, with details on the dimensions and inner structures. Images generated with Autodesk®'s CAD software Inventor Professional 2019.

The used metal mixers were designed in-house, using Autodesk®'s CAD software Inventor Professional 2019, with the same workflow as shown elsewhere [1,2]. MM1 had final dimensions of 100x33x45 mm LxHxW, and internal diameter of 1 mm and a volume of 2.37 mL. MM2 had final dimensions of 154x26.5x50 mm LxHxW, and internal diameter of 1 mm and a volume of 1.81 mL. The manufacturing was carried out by selective laser melting (SLM) at Anton Paar GmbH, with the same procedure shown in literature [1,2]. A close-up to MM1 and MM2 and an overview are this work are shown in **Figure S2**, while an overview of the mixers used in this work is given in **Figure S3**.

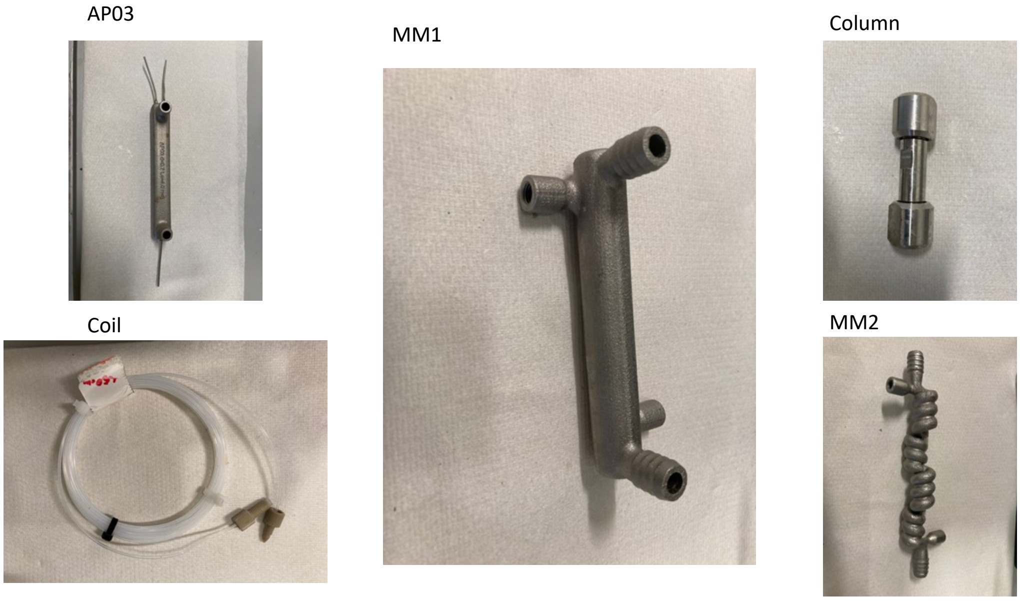

Figure S3 – Pictures of the mixers used in this work prior to the extractor.

3. Sensor readout of the $k_L a$ determination experiments

The measurements of the mass transfer rate of oxygen via $k_L a$ determination were carried out using the gassing out method as reported in the main article. Sensor readout values are reported in **Figure S4**.

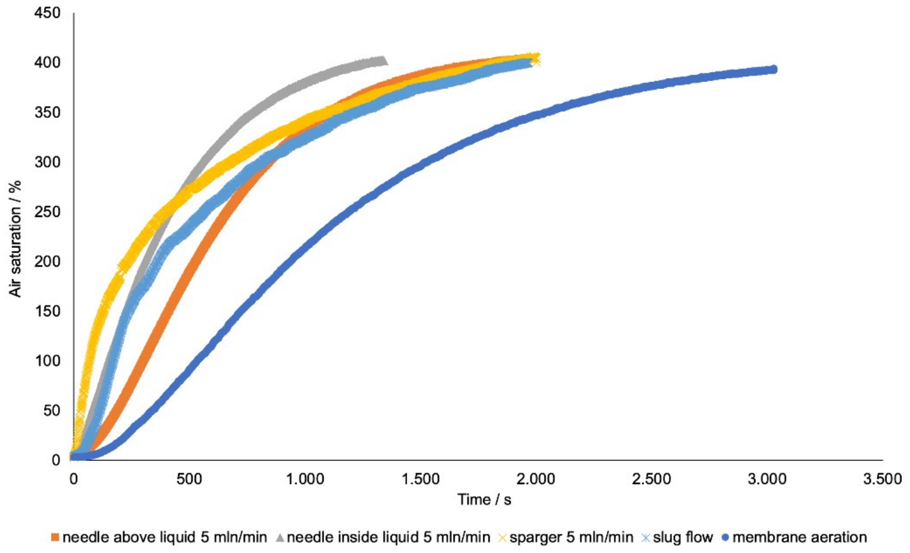

Figure S4 – Oxygen saturation versus time, recoded for different $k_L a$ measurements for different aeration modes.

4. Characterization of the residence time distribution in the 3D printed metal mixers

Characterization of the AP03 reactor by residence time distribution (RTD) was already carried out in a previous work.[2] The same procedure was used in this paper and will be briefly explained. A step input experiment was chosen to perform the RTD measurements, following a procedure from literature [3]. The experimental setup is shown in **Figure S5** (top). As a tracer solution, a mixture containing 0.008 v% anisole was used, dissolved in 12w% ethanol in water. The solvent and tracer solutions were filled in 2 syringe pumps and both lines were connected to a 6-way-valve, which was connected to the mixer's inlet. At the outlet of the mixer, the Avantes in-line flow cell with 10 mm path length detected the absorption of anisole between 268-274 nm while the baseline correction was recorded in the wavelength range of 500-506 nm, where anisole is not detectable. Before each experiment, a dark and light reference sample were taken. The experiment was carried out by generating a step signal when the 6-way-valve was switched to the inject position, to achieve a switch from no tracer to constant tracer concentration. After the absorbance value for the tracer reached a stable value, a step down signal was recorded by switching the valve back to load, so to only solvent and no tracer. The data was recorded by using the Avantes spectrometer software and then exported for evaluation in an Excel file after each run. The data was then evaluated in a separate Excel file to determine the cumulative and exit age distributions.

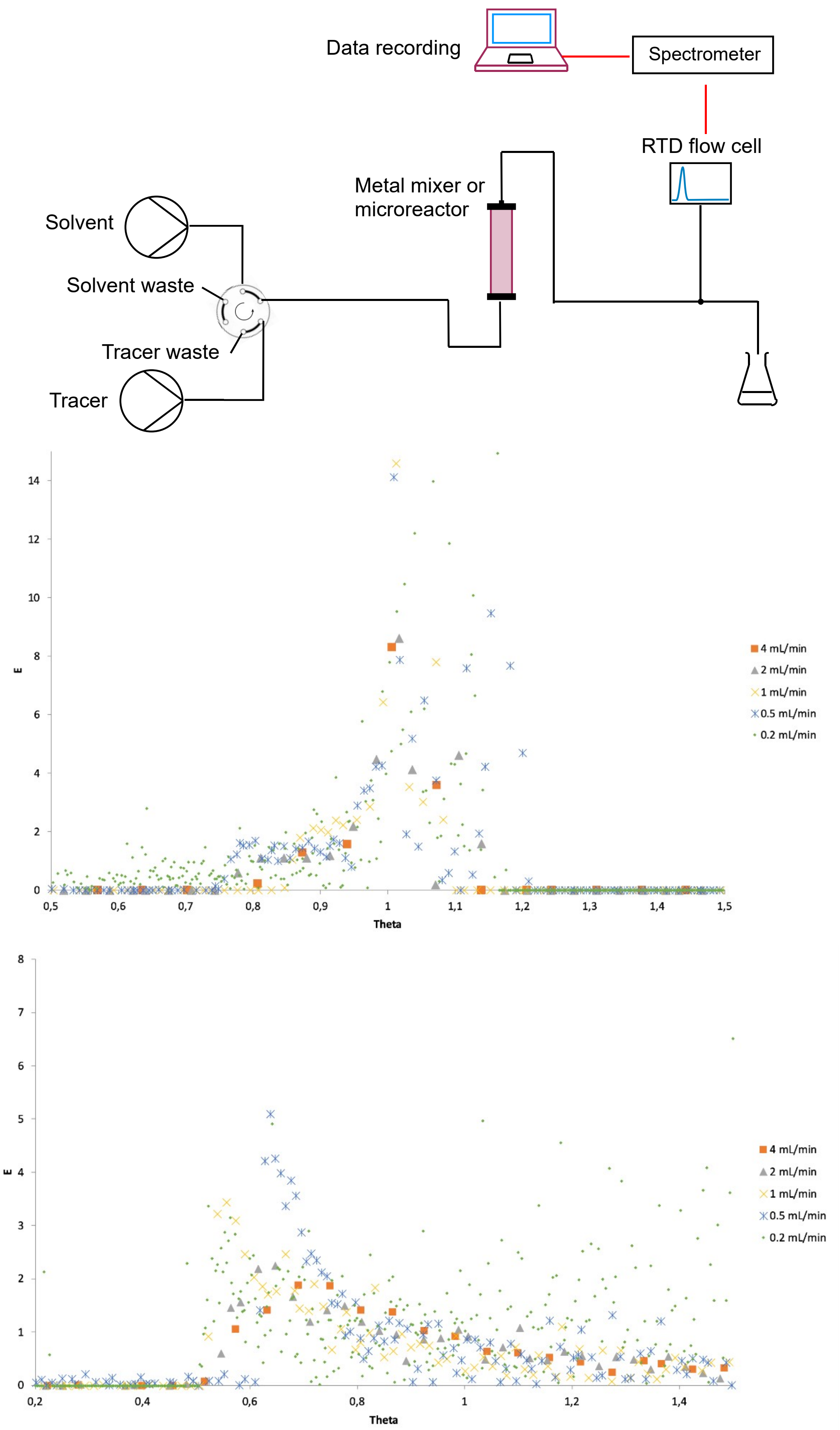

Figure S5- Top: Setup used for the RTD experiments. Middle: exit age distributions obtained for different flowrates in MM1. Bottom: exit age distributions obtained for different flowrates in MM2.

For each experiment, the Reynolds number (Re) was determined as:

$$Re = \frac{\rho \cdot u \cdot d_H}{\mu}$$

Where ρ and μ are, respectively the density and the dynamic viscosity of the fluid flowing through the channel, u is the flow velocity, and d_H is the inner channel diameter (1 mm for both mixers). The Bodenstein number (Bo) was also calculated in order to define whether the reactors showed a behavior of high or low backmixing and axial dispersion. In general, the Bo is defined as the ratio between the convective mass transfer and axial dispersion:

$$Bo = \frac{u \cdot L_{char}}{D_{ax}}$$

Where L_{char} is the characteristic length of the device and D_{ax} is the axial dispersion coefficient. For values of Bo above 100, the backmixing is considered low and similar to a plug flow reactor model; for low Bo values, instead, the backmixing is high and approaches an ideal CSTR model. Different methods are available to calculate Bo from the RTD curves, but in this work, the open-open model was chosen, as it assumes that the flow is dispersed along the length of the whole mixer, and it also provides an analytical solution to calculate Bo solely from the variance of the exit age distributions (E) curve. The E curves calculated for both mixers presented in this work with respect to the dimensionless time θ are reported in **Figure S5** (middle and bottom pictures) [3].

The results for the determination of the Bodenstein number are reported in **Table S1**. As visible from **Figure S5** (middle and bottom pictures), the E curves resulted in being noisy and affected by disturbances in the flow, especially at lower flow rates. This could be because, in this range, the extent of backmixing in the devices is very high, resulting in an irregular fluidic pattern that is mirrored by the irregular shape of the E curve and the presence of dispersed peaks [2,4]. Moreover, at low flow rates, the syringe pumps used show a pulsating behavior, which causes the tracer to not be injected with an ideal step [2,4]. The higher the flow rate, the more regular the E curves due to higher Re and superficial velocity, which give the chance to achieve secondary flow structures along the mixer, and therefore the axial dispersion is lower. The E curves were more symmetric and showed lower variance for MM1, which resulted in slightly higher values of Bo on average, indicating that MM1 shows a lower extent of backmixing compared to MM2.

Table S1 – Summary of the tested parameters and results for the RTD measurements for the two metal mixers presented in this work.

**Operating parameters**		**Metal mixer 1**		**Metal mixer 2**	
Flow rate [mL/min]	Re	Residence time [s]	Bo	Residence time [s]	Bo
4	75.013	32.07	21.64	31	10.41
2	37.5	63.87	22.81	56.16	11.19
1	18.75	109.3	33.16	107.8	5.17
0.5	9.37	247.6	9.69	189.4	10.74
0.2	3.75	768.2	9.42	546.6	8.92

4.1 Testing of possible hydrolysis of caprolactone in MM1

MM1 was the chosen mixer for the final setup, due to its improved mixing efficiency compared to the other mixers. In order to assess if caprolactone hydrolyzed in the mixer due to the enhanced mixing, the following straight-forward experiment was carried out. A solution of 200 mM of caprolactone in buffer was pumped through MM1 at 0.2 mL min^{-1}, and samples were collected every

15 minutes over the course of 2 hours. The samples were analyzed as during the process with GC-FID. The results, reported in **Figure S6**, showed that the concentration of caprolactone remained constant over the 2 hours of operation, indicating that no decomposition of the lactone due to hydrolysis or other unknown reactions took place.

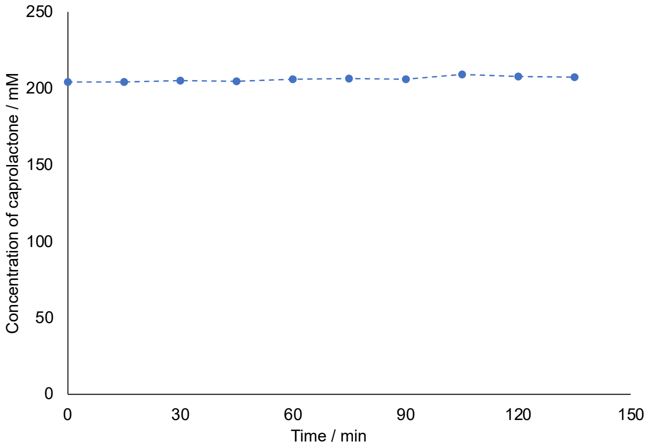

Figure S6 – Progression of the concentration of a 200 mM solution of caprolactone pumped through MM1. Samples were taken at the mixer outlet and measured with GC-FID, in order to test the possible decrease of the monomer concentration as a consequence of lactone hydrolysis.

5. Characterization of the residence time distribution in the packed tubular reactor

The tubular reactor packed with the immobilized CAL-B was also characterized via RTD, with a similar procedure mentioned above. However, it was noticed that anisole interacts with CAL-B, most probably due to absorption on the support's surface. Therefore, the tracer was changed to naphthalene, which was dissolved in 96 % ethanol. The operating procedure remained the same. The reactor, as packed for a continuous polymerization experiment, was connected to the setup shown in **Figure S5**. Then, the flowrate was set to the same as for the continuous experiments (0.1 mL min^{-1}) and the step up RTD experiment was carried out. The results for the exit age distribution, shown in **Figure S7**, show again an E curve with large variance, irregular shape, and presence of dispersed peaks at the end of the curve. This results in high backmixing, which is also mirrored by the low value of Bo (see **Table S2**). The residence time obtained was 11.6 min, which indicates that the reactor has an internal volume of 1.15 mL and a void fraction of 0.46.

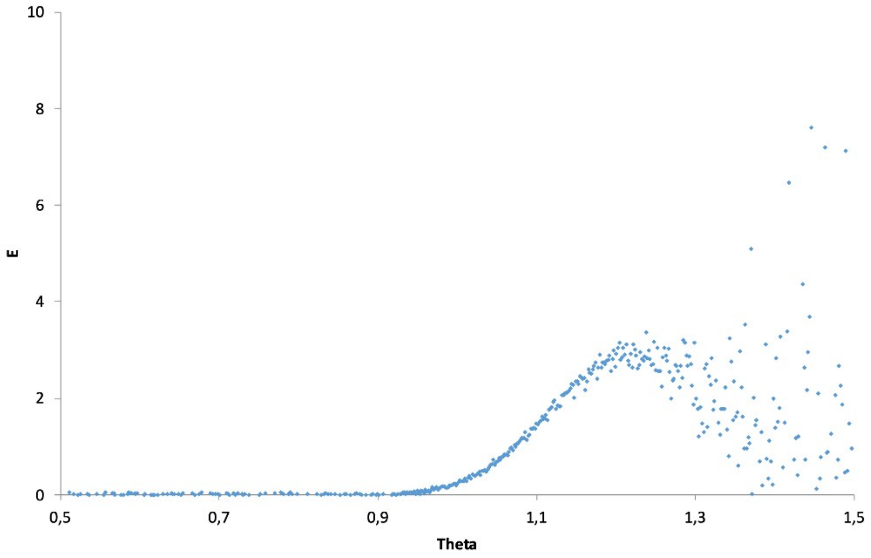

Figure S7 – Exit age distribution obtained for the polymerization reactor at 0.1 mL/min.

Table S2 - Summary of the RTD results for the polymerization reactor.

**Operating parameters**		**Reactor**	
Flow rate [mL/min]	Re	Residence time [min]	Bo
0.1	4.1	11.6	16.7

6. GC method and calibration curves

Table S3 – Details on the GC method used in this work.

Column parameters	Column	Optima 5-MS
	Film thickness	0.25 µm
	Length	30 m
	Inner diameter	320 µm
	Stationary phase	5 % Diphenyl – 95 % Dimethylpolysiloxane
Autosampler and injection port	Injection volume	1 µL
	Injection temperature	230 °C
	Carrier gas	N_2
	Total flow	21 mL/min
	Column flow	1 mL/min
	Split ratio	20

<table>
<tr><td rowspan="1">Temperature program</td><td></td><td><table><tr><td>Rate [°C/min]</td><td>Temp. [°C]</td><td>Hold [min]</td></tr><tr><td>---</td><td>60</td><td>5</td></tr><tr><td>10</td><td>200</td><td>3</td></tr></table></td></tr>
<tr><td rowspan="4">FID detector</td><td>Temperature</td><td>320 °C</td></tr>
<tr><td>Sampling rate</td><td>12.5 pts/s</td></tr>
<tr><td>H_2 flow</td><td>40 mL/min</td></tr>
<tr><td>Air flow</td><td>400 mL/min</td></tr>
<tr><td rowspan="3">Retention time</td><td>Substrate
Side product</td><td>Cyclohexanol: 7.32 min
Cyclohexanone: 7.45 min</td></tr>
<tr><td>Product</td><td>Caprolactone: 12.42 min</td></tr>
<tr><td>Initiator</td><td>3-phenylpropanol: 13.81 min</td></tr>
</table>

Samples were taken as described in the main article, and measured with GC-FID according to the method described in **Table S3**. A calibration curve was determined for all compounds, with 7 points (between 2 and 200 mM) and $R^2 > 0.98$. For the samples of the BVMO reaction and the aqueous sample of the continuous extraction with the SEP-10, the calibration for caprolactone, cyclohexanone and cyclohexanol was carried out as follows, in order to take into account the partial loss of caprolactone during extraction. A stock solution of 200 mM of each compound was prepared in the same buffer as the reaction, and it was diluted it in buffer to achieve the multiple calibration points (namely 200, 150, 100, 50, 25, 10 and 2 mM). Out of these solutions, 200 µL were sampled and treated as the samples during the process, therefore 600 µL of ethyl acetate were added and samples were vortexed for 1 min. Afterwards the organic solvent was removed in a separate vial for drying over $MgSO_4$, with subsequent centrifugation and transfer into GC vials. With this method, we determine the actual amount of caprolactone that can transfer into ethyl acetate from a buffered solution under different known starting concentrations, and by using the obtained calibration curve, we can therefore determine the concentration in the aqueous phase for other concentrations in the range. Calibration slopes [GC area/mM]: cyclohexanol 480.38, cyclohexanone 537.14, caprolactone extracted with ethyl acetate 411.98, caprolactone diluted in toluene 996.69, caprolactone diluted in CPME 839.25, 1-phenyl-propanol 1626.95. The concentrations for each compound in the samples were calculated from the peak areas and the calibration slopes.
Typical chromatograms are reported in **Figure S8**.

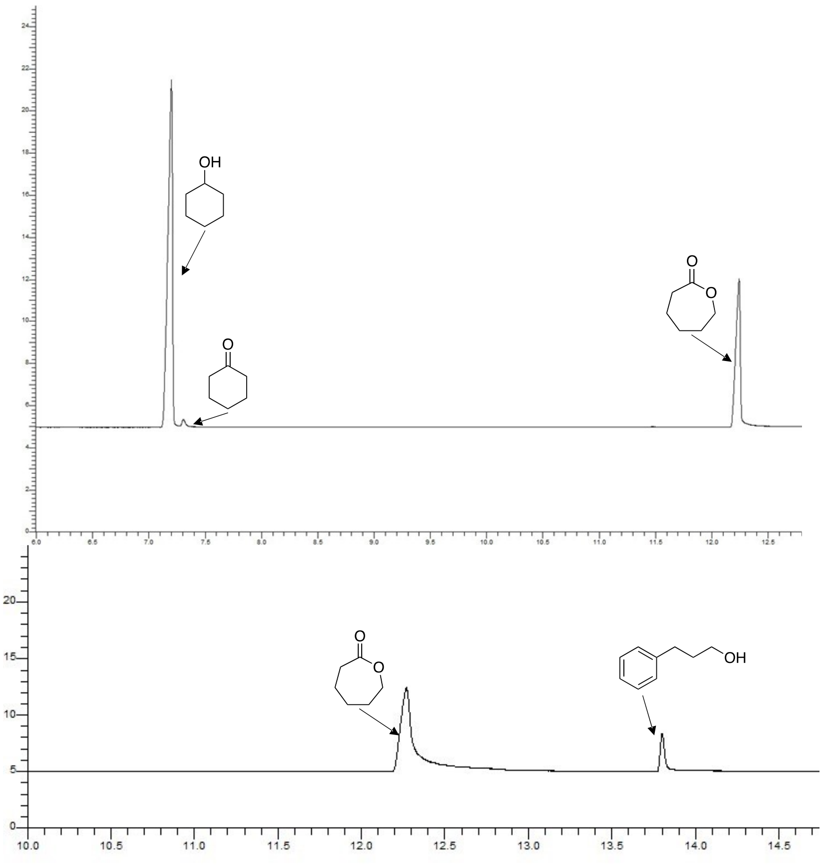

Figure S8 – Typical GC-FID chromatograms. Top: Sample GC chromatogram recorded for the batch biocatalytic cascade to produce caprolactone from cyclohexanol. Bottom: Sample chromatogram recorded from a sample taken at the outlet of the polymerization reactor containing both caprolactone and 2-phenylpropanol.

7. ^{1}H-NMR spectra

The following protocol was followed to calculate monomer conversion and average molecular weight from ^{1}H NMR spectra for the ROP of caprolactone, according to what was proposed in literature [5,6]. **Figure S9** shows the NMR spectra obtained for the isolation of caprolactone from the biocatalytic step. **Figure S10** shows the NMR spectra of caprolactone, precipitated in MeOH and filtrated after a flow experiment in toluene with 2 M starting concentration of caprolactone. **Figure S11** reports a sample NMR spectrum for the polymer produced from a flow experiment with 0.17 M starting concentration of caprolactone. For peak assignment, the guidelines from literature were used [5,6], and are reported in **Figure S10** and **Figure S11**. The shifts centered at 1.33 and 1.58 ppm were appointed to the PCL protons (CH_2- CH_3 and CH_4). Shifts at 1.68 and 1.78 ppm were assigned to the same type of protons but of the monomer ($CH_{2'}$- $CH_{3'}$ and $CH_{4'}$). CH_5 of PCL and $CH_{5'}$ of the monomer were assigned at 4 and 4.11 ppm. The signals of 1 and 1' protons overlap with the high-intensity signal of CH_3 of the toluene. Traces of leftover methanol are present as a peak at 3.4 ppm. The peak at 7.2 is assigned to $CDCl_3$.

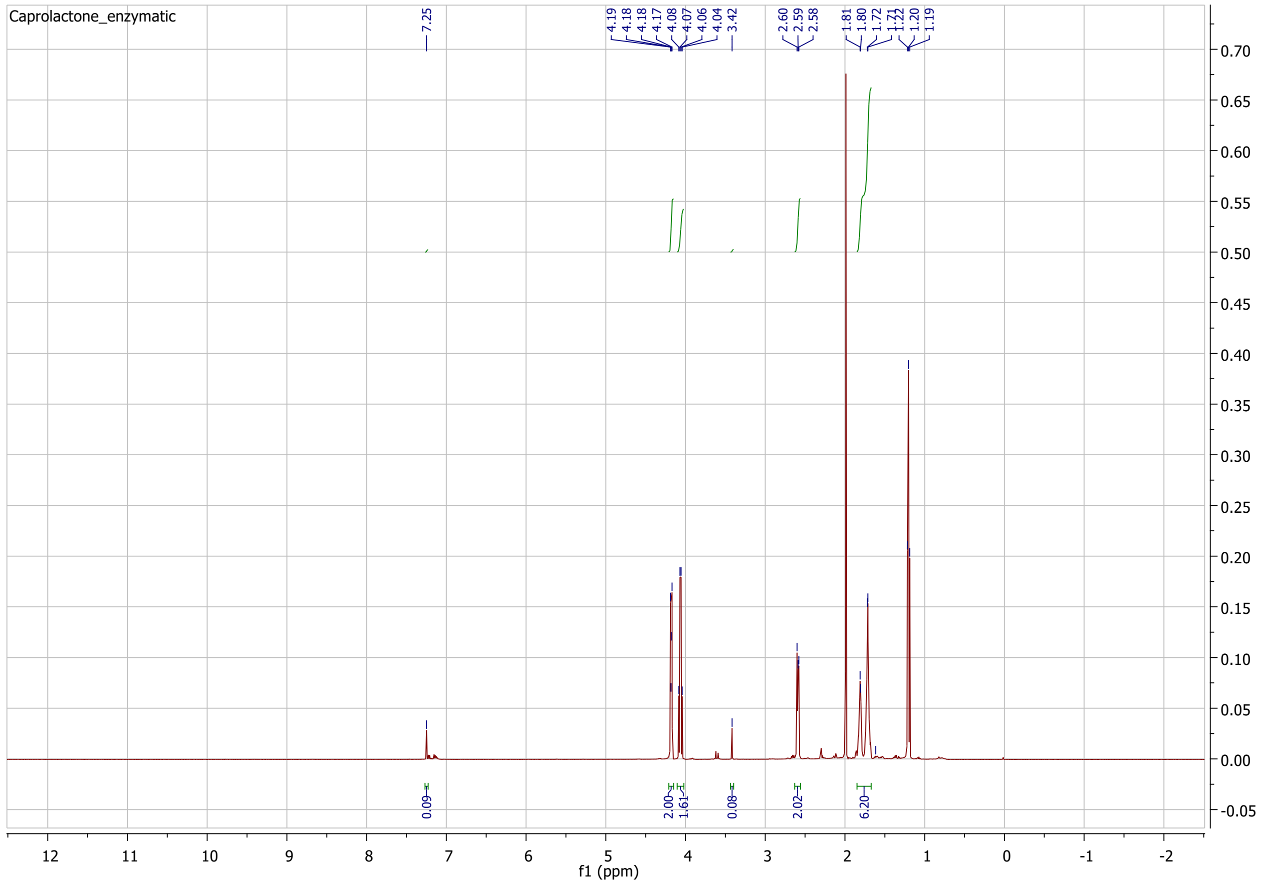

Figure S9 – NMR spectrum obtained for caprolactone isolated after the enzymatic cascade reaction. ^{1}H NMR (300 MHz, $CDCl_3$) δ 4.18 (d, J = 5.6Hz), 2.59 (s), 1.76 (d, J = 45.4 Hz).

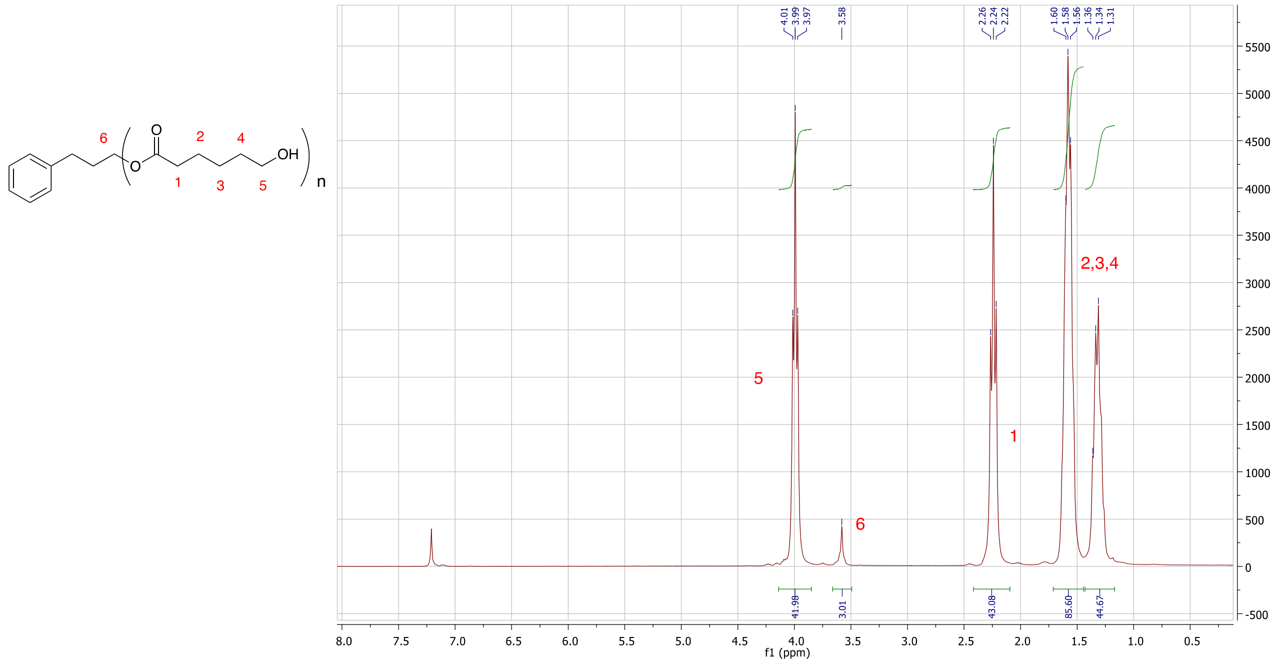

Figure S10 – NMR spectrum obtained for polycaprolactone, as isolated after the flow reaction with 2M starting material. 1H NMR (300 MHz, $CDCl_3$) δ 3.99 (t, J = 6.5 Hz), 3.58 (s), 2.24 (t, J = 7.4 Hz), 1.62 – 1.33 (m).

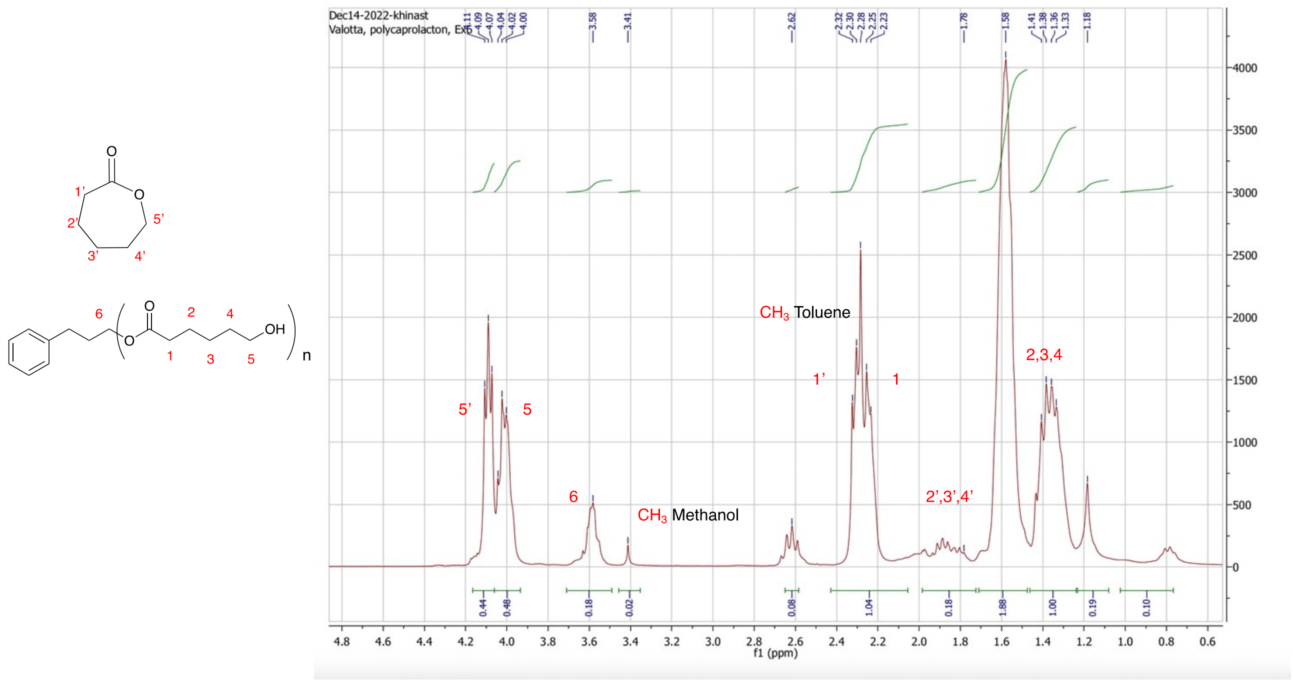

Figure S11 – Sample ^{1}H NMR spectrum obtained for a sample of polymer isolated and measured after a flow polymerization experiment with 170 mM starting concentration of caprolactone.

7.1 Calculation of the number-average molecular weight (M_n)

The M_n is related to the degree of polymerization (DP), or number of repeating units, which can be calculated by determining in ^{1}H NMR the spectra, the integral of the end-group of the polymer and the integral of the repeating chain unit of interest. In the case of the reaction at hand, the integral of the end group is calculated for the shift at 3.67 ppm, which represents the 2 protons of the O-CH_x bond between the repeating unit and the 3-phenyl propanol at the end of the polymer chain. This is correlated to the integration of the polymer CH_y protons of the repeating unit by the following equation[6]:

$$DP = \frac{[I(CH_y/y)]}{[I(O-CH_x)/x]}$$

Where y and x are protons of the polymer's repeating unit and of the bond with the initiator at the end chain, respectively. In the case of this reaction, the integration of the 2,3,4 peaks was considered (at 1.58 and 1.33 ppm) with y=6, and divided by the integral at 3.67 ppm with x=2. This value can be cross-checked by instead taking the value of the integration of the proton 5 (at 4 ppm) instead of 2,3,4, in this case y=2.
To then calculate the molecular weight, it is necessary to use the following equation [7]:

$$M_n = [DP * M(monomer)] + M(initiator)$$

Where M(monomer) = 114.14 g/mol and M(monomer)=136.19 g/mol.

8. Polymer characterization via GPC

For the final polymerization experiments, gel permeation chromatography (GPC) was used to determine the number average molecular weight (M_n) and weight average molecular weight (M_w). These values were determined for different process conditions, namely different starting concentrations of caprolactone (0.17 or 2 M), different setups (with or without molecular sieves),

and different extraction/reaction solvents (toluene or CPME). For each polymeric sample (see **Figure S12**), we report the molecular weight distributions in **Figure S13**. Moreover, the value for M_n was compared to that obtained by ^{1}H NMR to verify the validity of the used calculation method. For details on the GPC method, see the main article.

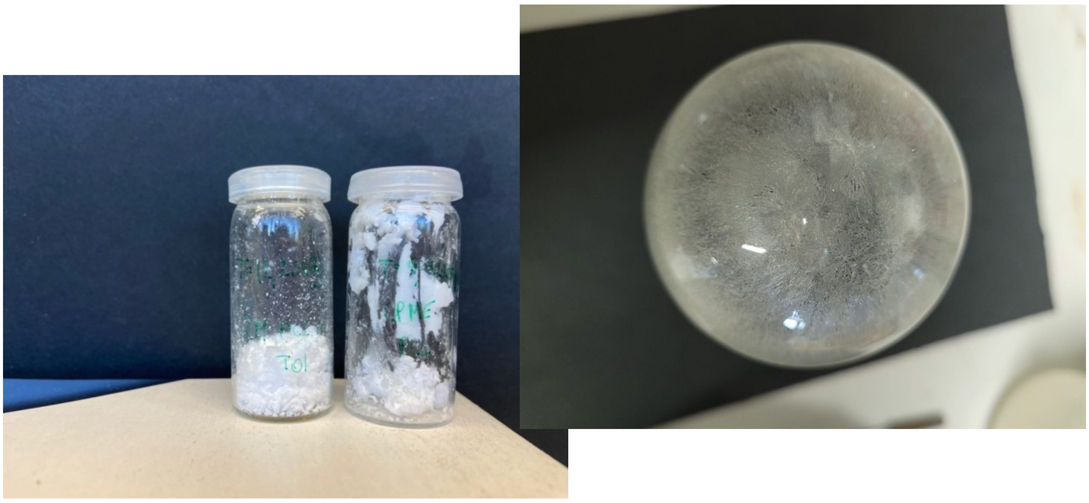

Figure S12 – Samples collected from the flow experiments starting with 2M of caprolactone (left) and with 0.17 M of caprolactone (right).

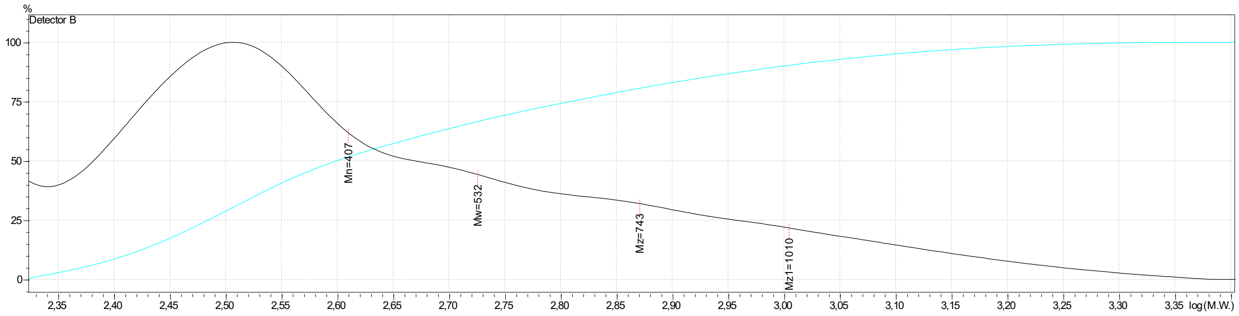

Molecular weight distribution – 0.17 M caprolactone in toluene

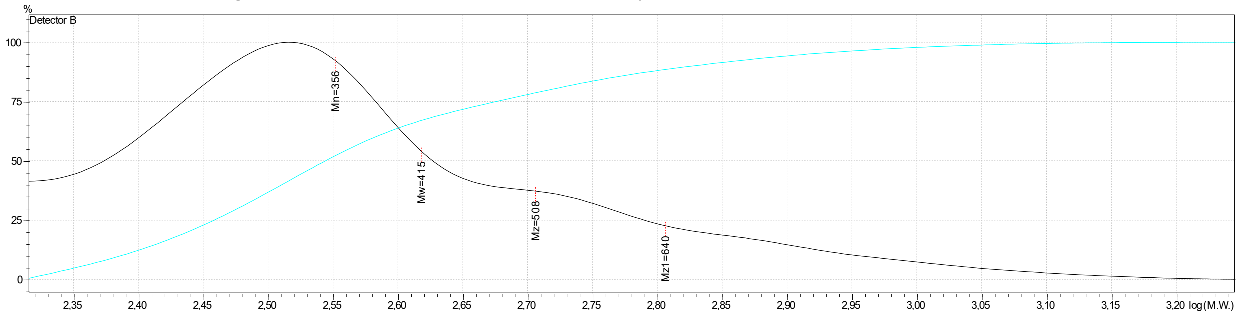

Molecular weight distribution – 0.17 M caprolactone in CPME

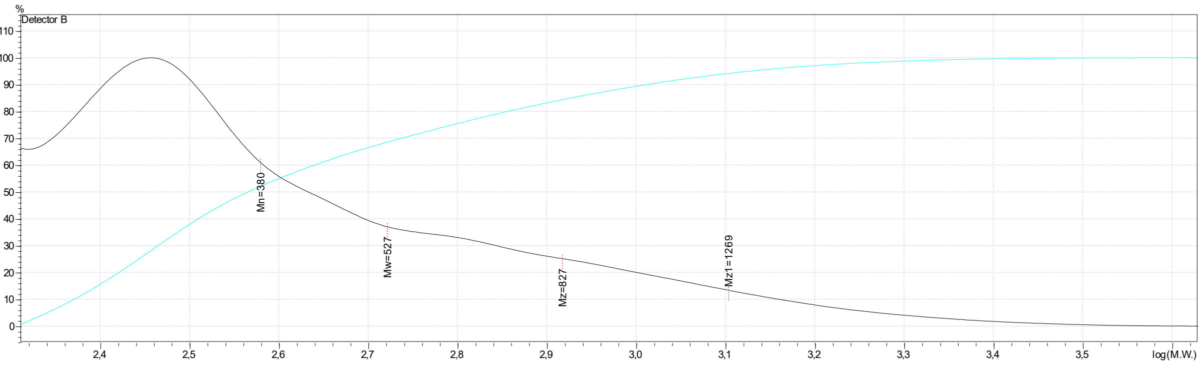

Molecular weight distribution – 0.17 M caprolactone in toluene, molecular sieves

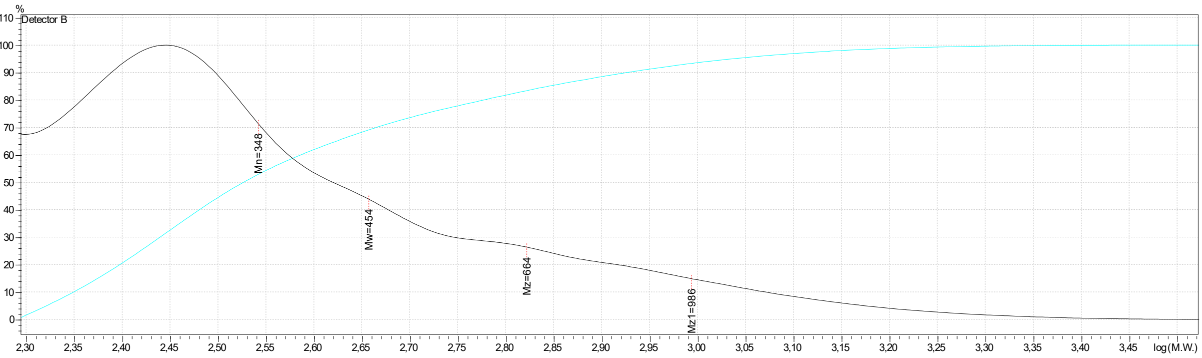

Molecular weight distribution – 0.17 M caprolactone in CPME, molecular sieves

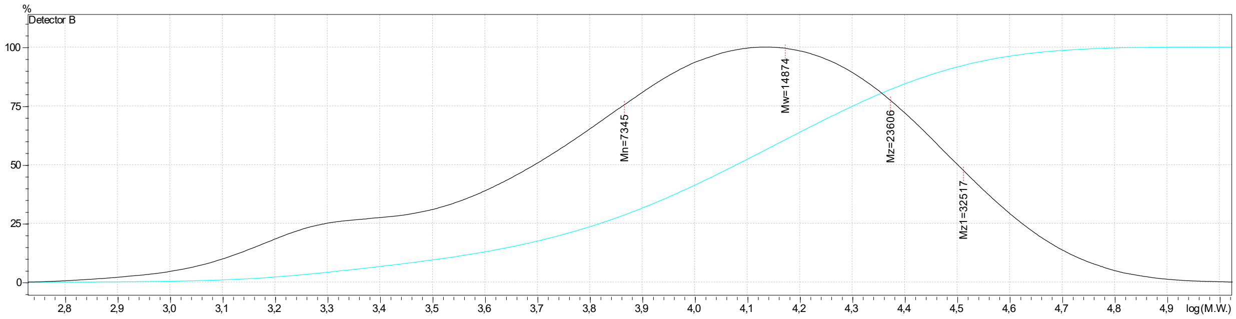

Molecular weight distribution – 2 M caprolactone in toluene

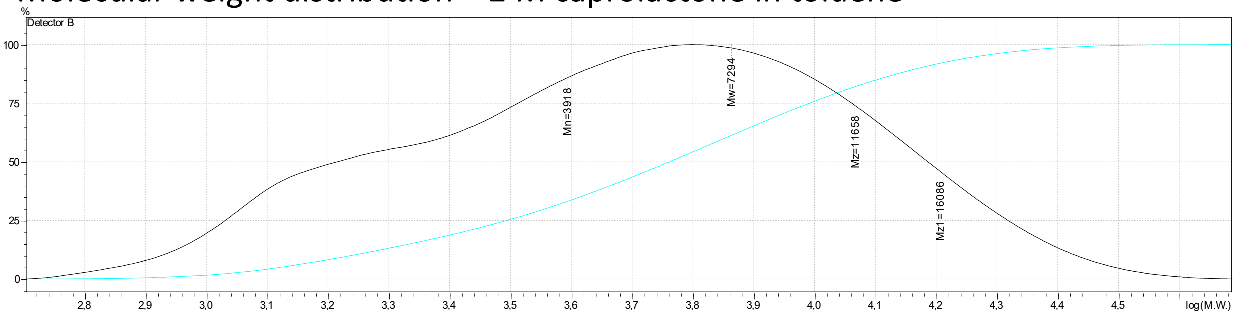

Molecular weight distribution – 2 M caprolactone in CPME

Figure S13 – Molecular weight (M_w) distribution curves recorded via GPC for different polymer samples produced starting with different concentrations of caprolactone (0.17 or 2M) and different extraction solvents (toluene or CPME).

9. References

1. Maier, M.C.; Lebl, R.; Sulzer, P.; Lechner, J.; Mayr, T.; Zadravec, M.; Slama, E.; Pfanner, S.; Schmölzer, C.; Pöchlauer, P.; et al. Development of Customized 3D Printed Stainless Steel Reactors with Inline Oxygen Sensors for Aerobic Oxidation of Grignard Reagents in Continuous Flow. *Reaction Chemistry & Engineering* **2019**, *4*, 393–401, doi:10.1039/C8RE00278A.
2. Maier, M.C.; Valotta, A.; Hiebler, K.; Soritz, S.; Gavric, K.; Grabner, B.; Gruber-Woelfler, H. 3D Printed Reactors for Synthesis of Active Pharmaceutical Ingredients in Continuous Flow. *Organic Process Research & Development* **2020**, *24*, 2197–2207, doi:10.1021/acs.oprd.0c00228.
3. Levenspiel, O. *Chemical Reaction Engineering*; 3rd ed.; John Wiley & Sons: New York, 1999; p. 686; ISBN 0-8176-4055-X.
4. Valotta, A.; Maier, M.C.; Soritz, S.; Pauritsch, M.; Koenig, M.; Brouczek, D.; Schwentenwein, M.; Gruber-Woelfler, H. 3D Printed Ceramics as Solid Supports for Enzyme Immobilization: An Automated DoE Approach for Applications in Continuous Flow. *Journal of Flow Chemistry* **2021**, *11*, 675–689, doi:10.1007/s41981-021-00163-4.
5. Bhangale, A.S.; Beers, K.L.; Gross, R.A. Enzyme-Catalyzed Polymerization of End-Functionalized Polymers in a Microreactor. *Macromolecules* **2012**, *45*, 7000–7008, doi:10.1021/ma301178k.
6. Adhami, W.; Bakkour, Y.; Rolando, C. Polylactones Synthesis by Enzymatic Ring Opening Polymerization in Flow. *Polymer* **2021**, *230*, 124040.
7. Izunobi, J.U.; Higginbotham, C.L. Polymer Molecular Weight Analysis by 1H NMR Spectroscopy. *Journal of Chemical Education* **2011**, *88*, 1098–1104, doi:10.1021/ed100461v.
